# Comprehensive Metabolite Profile Uncovers the Bioactive Components, Antioxidant and Antibacterial Activities in Wild Tree Peony Leaves

**DOI:** 10.3390/ijms241310609

**Published:** 2023-06-25

**Authors:** Ziwei Xin, Weizong Yang, Lixin Niu, Yanlong Zhang

**Affiliations:** College of Landscape Architecture and Arts, Northwest A&F University, Xianyang 712100, China; xinziwei2020@nwafu.edu.cn (Z.X.); yangweizong@nwafu.edu.cn (W.Y.)

**Keywords:** tree peony, newly grown leaf, bioactive components, antioxidant activity, antibacterial activity

## Abstract

Tree peonies (*Paeonia* Section *Moutan*)—including nine wild species, which belong to subsections *Vaginatae* and *Delavayanae*—are economically important plants with ornamental, nutritional, and medicinal applications. In this study, for the first time, we determined the bioactive components and antioxidant activities and antibacterial activities of the newly grown leaves of nine wild tree peony species (WTPS). A total of 276 bioactive components were identified through non-targeted metabolomics; more than 80% of the 276 metabolites identified are terpenoids and flavonoids. A total of 42 differential metabolites were quantitatively determined. The main differential metabolites were Paeoniflorin, Luteoloside, Hyperin, Apigenin-7-glucoside, Rhoifolin, and Cantharidin. Such a high terpenoid and flavonoid content of the leaf extracts renders them as species with strong antibacterial capacities, and most of the bacteria tested showed greater sensitivity derived from the members of subsection *Vaginatae* than those of subsection *Delavayanae*. All WTPS have significant antioxidant activity; this activity is attributed to high levels of the total phenolic content (TPC) and total flavonoid content (TFC), of which, among the nine WTPS, *P. lutea* has the strongest antioxidant capacity. Our results provided a theoretical basis for the in-deep application of tree peony leaves for food, medical, and pharmaceutical industries.

## 1. Introduction

**Figure 1:**
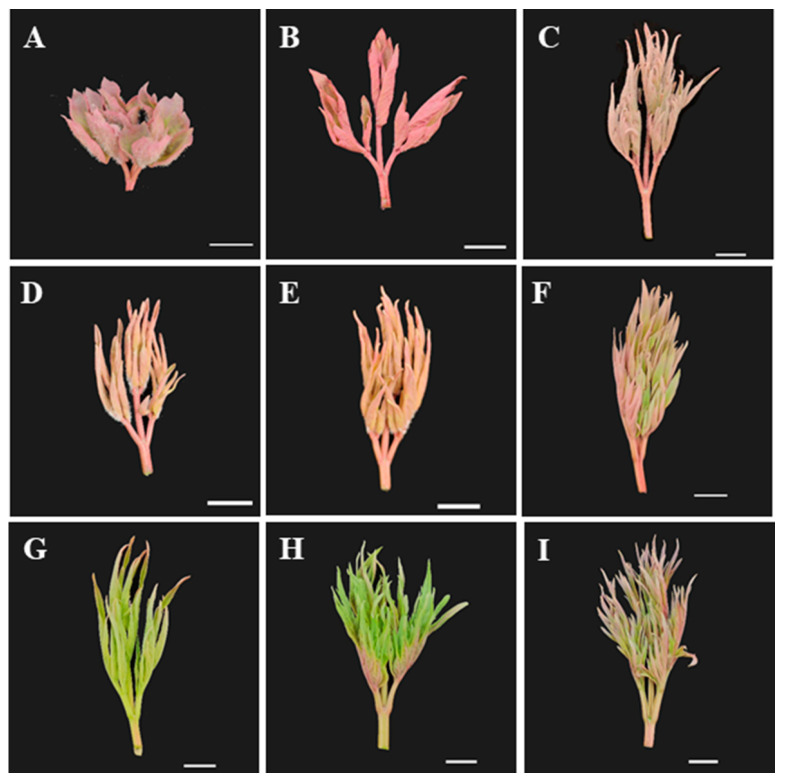
The newly grown leaves of nine WTPS. (**A**) *Paeonia jishanensis*. (**B**) *P. qiui*. (**C**) *P. decomposita*. (**D**) *P. ostii*. (**E**) *P. rockii*. (**F**) *P. lutea*. (**G**) *P. delavayi*. (**H**) *P. ludlowii*. (**I**) *P. potaninii.* Bars = 10 mm.

For millennia, the plant genus *Paeonia* has been recognized worldwide as an important group of ornamental and medicinal plants [1]. The genus *Paeonia* is further subdivided into sections and subsections. For instance, tree peonies belong to section *Moutan*. Grouped under this section are the nine wild species (Figure 1) *P. jishanensis*, *P. qiui*, *P. decomposita*, *P. ostii*, and *P. rockii* belonging to subsection *Vaginatae* and the other four species (*P. lutea*, *P. delavayi, P. potaninii*, and *P. ludlowii*) belonging to subsection *Delavayanae* [2]. In 2018, the tree peony was introduced in the European Pharmacopoeia Supplement 9.4 for the first time for its medicinal value, including anti-diabetic, anti-atherosclerotic, anti-cancer, anti-inflammatory, cytoprotective, cardioprotective, and hepatoprotective properties. Moreover, the importance of tree peonies in cosmetic production was also highlighted [3,4,5].

Plants are important sources of several valuable secondary metabolites [6,7] which lead to their biological activities. In recent years, plant extracts have become widely used as antibacterial and antioxidant agents for medical, pharmaceutical, cosmetic, and food applications [8,9,10,11]. Such a high demand for medicinal plant extracts is brought about by the inefficacy of conventional antibiotics in treating infections caused by drug-resistant bacteria [12]. Bacteria have been shown to use signaling systems such as quorum sensing to regulate cellular interactions via small chemical signaling molecules [13]. On such basis, monoclonal antibodies targeting the pathogens and their components, as well as the application of nanotechnology, have emerged as new antimicrobial strategies [14,15]. However, plant extracts can be used more quickly and simply as additives in food or pharmaceuticals. Thus, plant extracts are now being considered as potential alternative sources of antimicrobial substances [16].

Tree peonies are economically important plants: their fruits and oil are edible, their flowers serve as ornaments, and their roots have well-known medicinal properties [17,18,19,20,21]. Hence, most research on peonies focuses on their roots, flowers, fruits, and oil [22,23,24]. The bioactive compounds and bioactivities of their leaves are largely unknown. Previous studies on peony leaves have reportedly focused on their mature leaves, which are in full bloom period [9]; however, to our knowledge, there is almost no research on newly grown tree peony leaves. It is well known that the nutrients required for the sprouting of tree peony leaves are obtained from the roots, so there is a certain similarity in the types of secondary metabolites between the roots and leaves of tree peony [25]. As the leaves develop, the species and content of secondary metabolites in them decrease. This illustrates the necessity and importance of research on newly grown leaves of tree peony, which, in addition, are more competitive than mature leaves for both tea and medicinal use.

In this study, we comprehensively analyzed and assessed the bioactive compounds and bioactivities—antioxidant and antibacterial—of the solvent extracts derived from the newly grown leaves of wild tree peonies. Our findings indicate that newly grown tree peony leaves may serve as alternative sources of antioxidant and antibacterial substances, which have potential applications in medical, pharmaceutical, cosmetic, and food industries.

## 2. Results and Discussion

### 2.1. Total Phenolic Content of the Leaves of Nine WTPS

**Figure 2:**
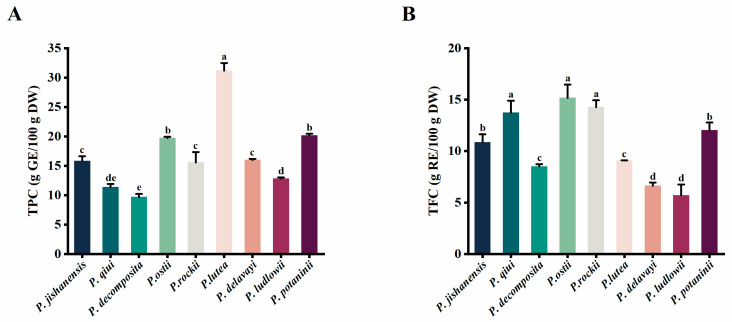
(**A**) The TPC of the newly grown leaves of nine WTPS. (**B**) The TFC of the newly grown leaves of nine WTPS. Column and error bar represents mean values and standard deviation (*n* = 3). Different letters in each column (a–e) denote significant differences (*p* < 0.05), as determined by Tukey’spost-test. Different colors in each column indicate different wild species of tree peonies.

The TPC of the newly grown leaf extracts derived from nine WTPS had significant differences (Supplementary Table S2 and Figure 2A), ranging from 9.68 to 31.18 g GE/100 g DW. The leaves of *P. lutea* had the highest TPC (31.18 g GE/100 g DW), while those of *P. decomposita* had the lowest (9.68 g GE/100 g DW). The TPC of *P. ludlowii* (12.82 g GE/100 g DW), which was relatively low, is almost similar to the previously reported TPC of *P. rockii* leaves [26]; such a similarity proves the reliability of our experimental results. The TPC of peony newly grown leaves was significantly higher than that of peony fruits, stamens, and petals [27,28,29]. Therefore, the leaves of wild tree peonies are rich in phenols, indicating that peony leaves have a great potential for future product development.

### 2.2. Total Flavonoid Content of the Leaves of Nine WTPS

The TFC of the leaves of the nine WTPS had significant differences (Supplementary Table S2 and Figure 2B), ranging from 5.70 to 15.16 g RE/100 g DW. The newly grown leaves of *P. ostii* had the highest TFC (15.16 g GE/100 g DW), while those of *P. ludlowii* had the lowest (5.70 g GE/100 g DW). The results revealed that the TFC of the wild tree peony leaves was significantly higher than the previously reported TFC of cultivated peony species [30], indicating that the leaves of wild tree peonies have a higher development and utilization value.

### 2.3. LC-MS Non-Targeted Metabolome Analysis of the Leaves of Nine WTPS

**Figure 3:**
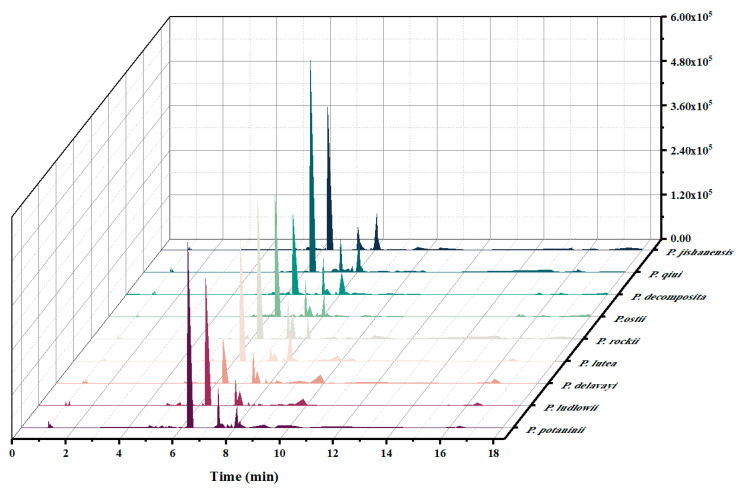
Fingerprint of biochemical compounds extracted from the newly grown leaves of nine WTPS.

**Figure 4:**
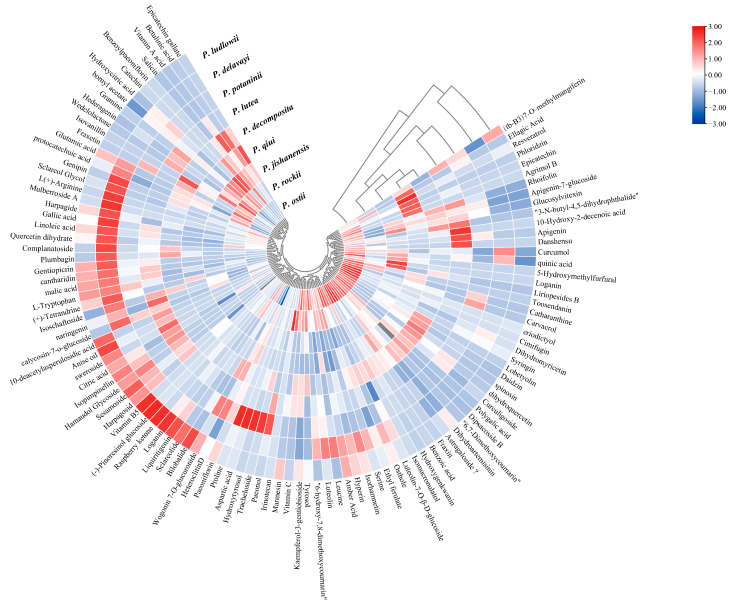
Heat map of the log2 fold change of significant changes among the main differential metabolites from the newly grown leaves of nine WTPS.

In this study, we analyzed and compared the metabolome of newly grown leaves of the nine wild tree peonies. The bioactive composition of the extracted are shown in Supplementary Table S3, Figure 3 and Figure 4. A cumulative total of 276 bioactive components were identified. The number of compounds identified in the two subsections was almost similar: 209 in subsection *Vaginatae* and 185 in subsection *Delavayanae*. *Paeonia qiui*, belonging to subsection *Vaginatae,* had the highest number of compound species (113 species), while *P. delavayi,* belonging to subsection *Delavayanae,* had the lowest number (85 species).

**Figure 5:**
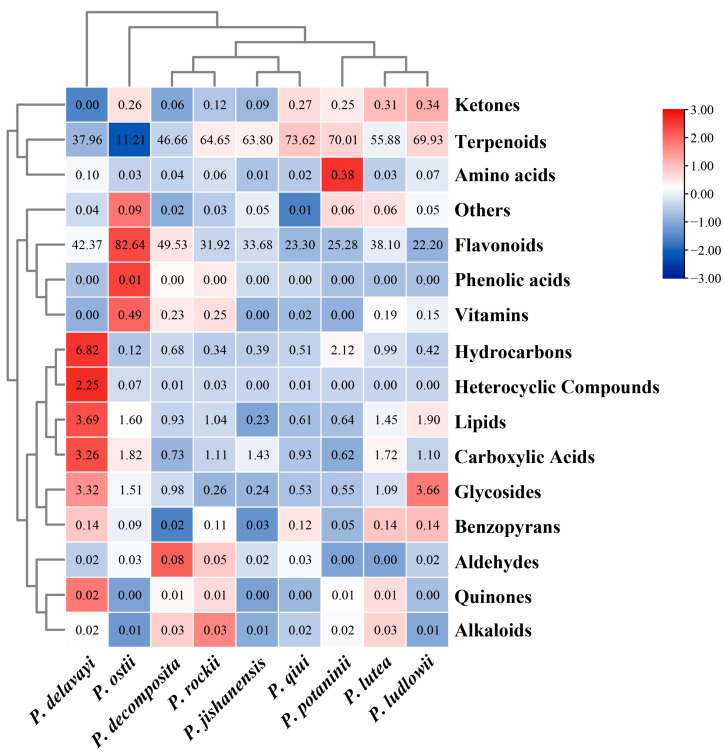
Heat map of the log2 fold change of significant changes among the different biochemical classes in the newly grown leaves of nine WTPS based on the relative content (%) of bioactive compounds. The data were normalized by row.

These bioactive components can be classified into the following categories based on their metabolite type: terpenoids, flavonoids, lipids, hydrocarbons, glycosides, carboxylic acids, amino acids, phenolic acids, benzopyrans, quinones, alkaloids, vitamins, heterocyclic compounds, aldehydes, ketones, and others. Most of the bioactive compounds were terpenoids and flavonoids, accounting for more than 80% of the total bioactive components (Supplementary Table S4 and Figure 5). This result on wild peony species is consistent with those of previous findings of cultivated peony species [31]. The data shown in figure legend of Figure 5 (−3 to 3) were logarithmically transformed by taking the log value (log2). In order to better show in Figure 5 in which wild tree peony species the total content of each different class of substances is highest, the data were normalized by row scale. Therefore, in each row, a redder color indicates a larger value, and the shade of the color is determined by the size of the difference between the data values in that row. Through t-test, different metabolites were screened based on variable importance in projection (VIP) scores > 1 and *p* < 0.05 to clarify the specific differences in chemical components in the newly grown leaves of nine WTPS. A total of 42 compounds with significant differences were screened, including 14 flavonoids, 6 terpenoids, 6 carboxylic acids, 5 glycosides, and 12 other metabolites (Supplementary Table S5). We quantitatively determined these differential metabolites; results showed six metabolites with contents exceeding 50 mg/mL, namely Paeoniflorin (24.42 to 233.97 mg/mL), Luteoloside (1 to 219.94 mg/mL), Hyperin (1.34 to 103.12 mg/mL), Apigenin-7-glucoside (0.16 to 103.29 mg/mL), Rhoifolin (0.08 to 57.73 mg/mL), and Cantharidin (0 to 76.14 mg/mL).

**Figure 6:**
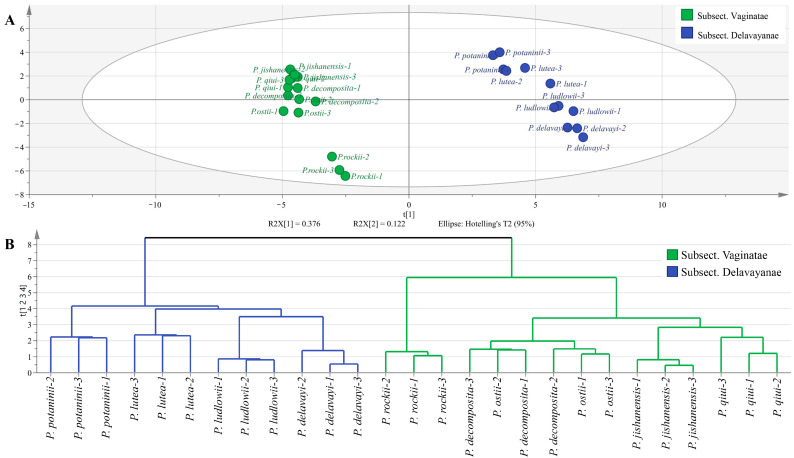
(**A**) The PCA score scatter plot; (**B**) The HCA plot of the PCA.

**Figure 7:**
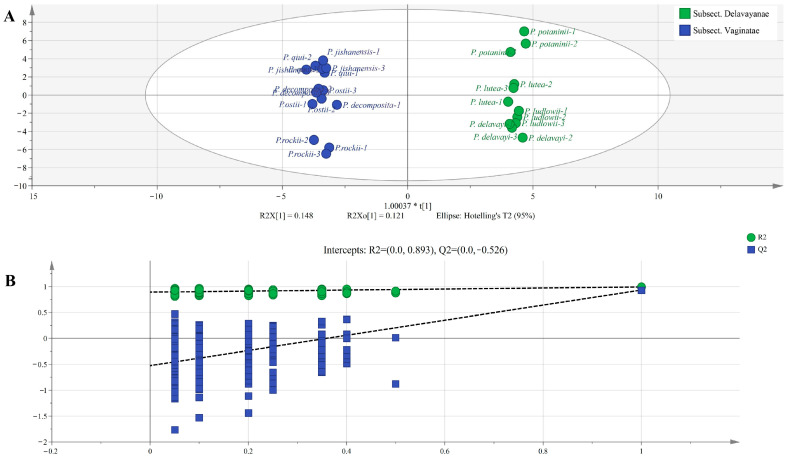
(**A**) The OPLS-DA score scatter plot; (**B**) Displacement check diagram for subsections *Vaginatae* and *Delavayanae*.

To further evaluate the differences in metabolites present among the newly grown leaves of the nine WTPS and to determine their relationship with subsections *Vaginatae* and *Delavayanae*, multivariate statistical analyses were performed with SIMCA 14.1 (Figure 6 and Figure 7). Our research team also used this method for multivariate analysis of metabolites of peony pods and seeds during development in our previous study, which proved the reliability of the analysis method [29]. The samples were comprehensively analyzed using unsupervised PCA statistical tools. As shown in Figure 6A, triplicate samples of the same wild tree peony species were clustered together, indicating that the metabolomics approach is sufficiently reproducible. The sum of the principal components accounted for 63.1%, indicating the reliability of the grouping. The PCA score scatter plot showed a clear separation between subsections *Vaginatae* and *Delavayanae*, indicating that the two subsections possess substantially different biochemical profiles. As a clustering analysis method, HCA searches the optimal distribution of clusters using a hierarchical structure. As shown in Figure 6B, the nine WTPS are clearly divided into two groups, and their grouping is consistent with the botanical subgroup classification: subsection *Vaginatae* and subsection *Delavayanae*. This classification result is consistent with that obtained by our research team in a previous compositional clustering of petals from nine WTPS [32]. Consequently, significant differences between the groups need further study.

In order to analyze the differences between groups, the OPLS-DA model with the supervisory function was used for analysis. The results of the OPLS-DA score plot (Figure 7A) showed that the three replicates of all samples were clustered together, while the samples of the subsections *Vaginatae* and *Delavayanae* were distributed on both sides of the 95% confidence interval. The obvious identification effect indicates a significant difference in the composition of the two subsections attributed to differential metabolites. The linear regressions of R^2^ and Q^2^ show a downward trend with a decrease in the replacement retention rate (Figure 7B), indicating that the replacement test has passed and the model is reliable.

### 2.4. Evaluation of Antioxidant Activity of the Leaves of Nine WTPS

As is well known, secondary metabolites in plants often have biological activity, and in order to bring the secondary metabolites in wild tree peony leaves into practical application value, we conducted activity validation on tree peony newly grown leaf extracts.

**Figure 8:**
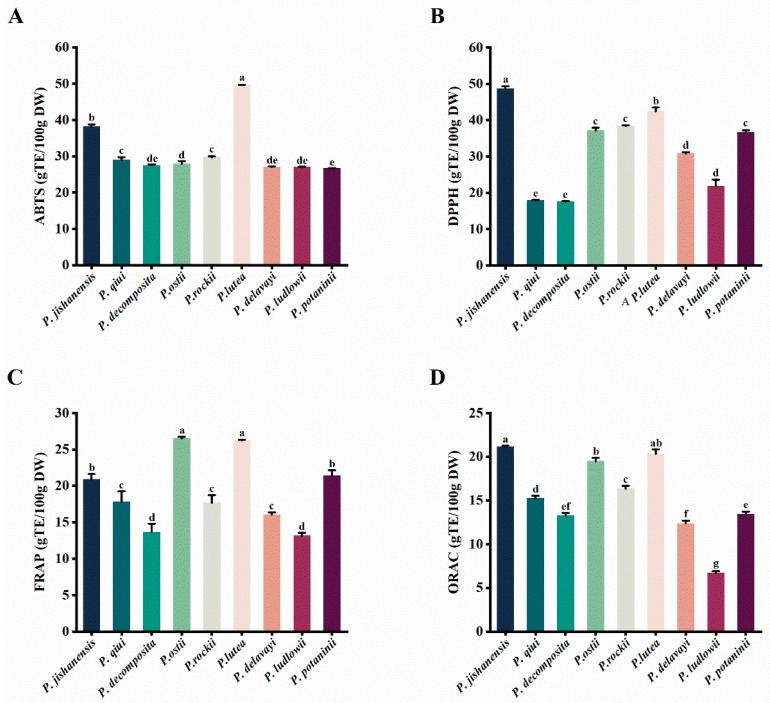
In vitro antioxidant activities of the newly grown leaves of nine WTPS. (**A**) ABTS^+•^ assays. (**B**) DPPH^•^ assays. (**C**) FRAP assays. (**D**) ORAC assays. Column and error bar represent mean values and standard deviation (*n* = 3). Different letters in each column (a–g) denote significant inter-species differences (*p* < 0.05), as determined by Tukey’s post-test. Different colors in each column indicate different wild species of tree peonies.

To more accurately evaluate the antioxidant performance of the samples, four methods (ABTS^+•^, DPPH^•^, FRAP, and ORAC) were employed to determine the antioxidant activity of the leaves of the nine WTPS (Supplementary Table S6 and Figure 8). The scavenging capacity of the leaves of nine wild species to ABTS^+•^ free radicals ranged from 26.72 to 49.62 g TE/100 g DW. Compared with the peony petals [33], newly grown peony leaves have a stronger antioxidant activity. Among the nine WTPS, *P. lutea* had the strongest antioxidant capacity (49.62 g TE/100 g DW), while *P. decomposita* had the weakest. Moreover, *P. jishanensis* had the highest (48.68 g TE/100 g DW) DPPH^•^ scavenging capacity followed by *P. lutea* (42. 39 g TE/100 g DW); *P. decomposita* had the lowest (17.59 g TE/100 g DW). Meanwhile, the previously reported DPPH^•^ value of peony roots was only 3574.47–2172.71 μmol TE/100 g DW [19]. Thus, peony leaves have a stronger antioxidant activity than peony roots. The FRAP results revealed that *P. ostii* had the strongest (26.51 g TE/100 g) iron-ion reduction ability, followed by *P. lutea* and *P. potaninii*. Compared with the *P. ostii* roots and the stamens of *P. ostii* [19,29], the newly grown leaves of *P. ostii* have stronger antioxidant capacity. The ORAC and FRAP activities of the newly grown leaves exhibited similar distribution patterns in several wild tree peony species. *Paeonia jishanensis* had the strongest antioxidant capacity (21.14 g TE/100 g DW), followed by *P. lutea* (20.32 g TE/100 g DW). On the other hand, *P. ludlowii* had the lowest scavenging capacity for ORAC (6.70 g TE/100 g DW), FRAP (13.14 g TE/100 g DW), and ABTS^+•^ (27.00 g TE/100 g DW) free radicals. Among the nine WTPS, *P. lutea* exhibited the strongest antioxidant capacity, followed by *P. jishanensis* and *P. ostii*; meanwhile, *P. ludlowii* and *P. decomposita* had the weakest capacity. Based on these results, the leaves of *P. lutea*, *P. jishanensis,* and *P. ostii* can be considered the first choice for the development of antioxidant products.

### 2.5. Evaluation of the Antibacterial Activity of the Leaves of Nine WTPS

Due to the widespread use of antibiotics, more and more bacteria are beginning to develop drug resistance [34]. Therefore, it is necessary to search for antibacterial active ingredients from plant extracts as novel antibacterial agents.

**Figure 9:**
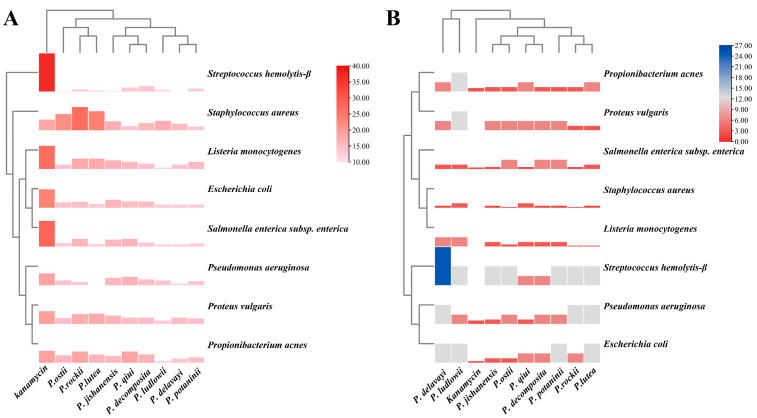
(**A**) The diameters of inhibition zones (mm) of eight bacteria tested in vitro after the application of leaf extracts derived from nine WTPS. (**B**) Minimum inhibitory concentrations (mg/mL) of leaf extracts derived from nine WTPS against eight bacteria tested in vitro.

The results of the agar diffusion test are presented in Figure 9B, Figures S1 and S2. The solvent extracts from the leaves of the nine WTPS showed significant inhibitory effects on all bacteria tested: the diameters of inhibition zones (DIZ) ranged from 10.83 to 26.50 mm. In contrast, after the application of kanamycin, the DIZ ranged from 18.17 to 36.60 mm. Meanwhile, methanol was unable to inhibit bacteria growth. Compared with the microbial growth inhibitory effect (10.65–17.5 mm) of newly grown peony leaf hydrosols [25], it is safe to conclude that the solvent extract from peony leaves exhibits a stronger antibacterial activity than that of peony leaf hydrosols. Compared to the antibacterial ability of solvent extracts of *P. suffruticosa* flower buds against food-borne pathogens such as *S. aureus*, *L. monocytogenes*, and *E. coli* reported previously (DIZ and MICs: 9–15 mm and 0.195–6.25 mg/mL, respectively), the antibacterial ability of wild tree peony leaves was clearly greater [35]. To more accurately assess the antibacterial activity of the newly grown peony leaves, MIC assay was also employed. The test and calculation results for MICs are presented in Figure 9A and Supplementary Table S7. The results revealed some differences, ranging from 0.78 to 25 mg/mL, in MIC values among the solvent extracts derived from the leaves of the nine WTPS. Compared with the previously reported antibacterial effect of the newly grown leaf hydrosols of *P. ostii*, the solvent extract from newly grown wild tree peony leaves produces a better antibacterial effect.

Among the bacteria tested, the newly grown leaf extracts demonstrated the strongest inhibitory activity against *Staphylococcus aureus*, followed by *Propionibacterium acnes*, *Listeria monocytogenes*, and *Salmonella enterica* subsp. *enterica*; however, the weakest inhibitory activity was demonstrated against *Streptococcus hemolytis-β*. Most of the bacteria tested were more sensitive to *P. jishanensis* and *P. rockii* leaf extracts, followed by *P. lutea*, *P. ostii*, and *P. qiui*. We observed that the members of subsection *Vaginatae* had more effective antibacterial properties than those of subsection *Delavayanae*. However, proving this requires further in-depth study. Nevertheless, we are certain that the leaf extracts of wild tree peonies have potential antibacterial effects, which deserves further research and development.

### 2.6. Multivariate Statistical Analysis

**Figure 10:**
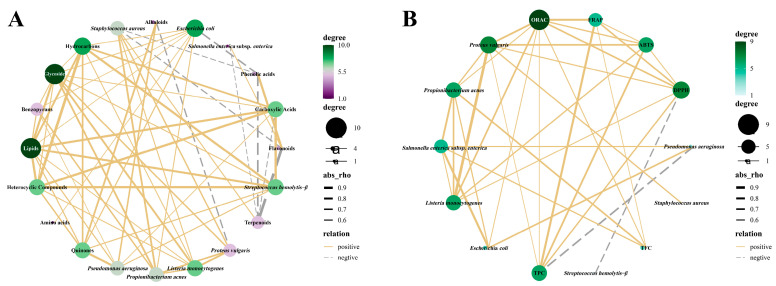
(**A**) Correlation network between bioactive components and antibacterial activities. (**B**) Correlation network between TPC, TFC, and bioactive activities.

Owing to the presence of bioactive compounds, tree peonies exhibit corresponding biological activities. Therefore, as shown in Figure 10, Pearson’s correlation analysis was used to reveal the potential relationship between the assayed bioactive compounds and the tested bioactivities.

As shown in Figure 10A, the antibacterial activity of wild tree peony leaves is significantly correlated with the bioactive compounds. It is noteworthy that terpenoids and flavonoids accounted for the largest proportion of substances; however, terpenoids and flavonoids were only significantly correlated with *Staphylococcus aureus* and *Proteus vulgaris*, respectively. The remaining six bacteria were correlated with multiple substances. Based on the results, the solvent extracts from wild tree peony leaves effectively inhibited the growth of *Staphylococcus aureus* and *Proteus vulgaris*. This indicates that, in addition to the synergistic effects of various substances, terpenoids and flavonoids alone contribute to antibacterial activity.

The biological activity of peony leaves was significantly correlated with TPC and TFC (Figure 10B), and TPC had a significant positive correlation with four antioxidant radical-scavenging activities (ABTS^+•^, DPPH^•^, FRAP, and ORAC). Moreover, there was no correlation between TPC and most test strains except *Proteus vulgaris*; thus, there is no significant correlation between TPC and antibacterial activity. TFC was strongly positively correlated with FRAP and ORAC radical scavenging activities; it was also strongly correlated with the inhibition of the growth of *Propionibacterium acnes* and *Salmonella enterica*. This indicates that the biological activity of newly grown wild tree peony leaves is inextricably correlated with high TPC and TFC.

## 3. Materials and Methods

### 3.1. Plant Materials

Wild tree peonies have been growing in the General Forestry Science and Technology Extension Station of the Forestry Department, Lanzhou City, Gansu Province for over 20 years since their introduction to their native habitats in 2001. We collected wild tree peony leaves from March to April 2021, the whole collection period was 8–10 days. All solvent extracts were pretreated with liquid nitrogen prior to use and then kept at −80 °C. Species identification was confirmed by Lixia He—a researcher from Gansu Province.

### 3.2. Sample Extraction

The leaves collected were pretreated using a vacuum freeze dryer; then, the dried leaves were ground into a powder. With the aid of a 40 kHz ultrasonic, a sample was extracted from 1 g of powdered leaves mixed with 15 mL of methanol for 30 min and then centrifuged at 12,500 rpm for 15 min [36]. Following the above steps, the extraction was repeated twice for the residue. After three extractions, the supernatant was mixed. The solvent extracts were filtered through a 0.22 μm membrane filter, and then stored at 4 °C until further use.

### 3.3. Chemicals

Water-soluble vitamin E (Trolox), 1,1-diphenyl-2-trinitrophenylhydrazine (DPPH), 2,2′-Azino-bis-(3-ethylbenzothiazoline-6-sulfonic acid), diammonium salt (ABTS), and 2,4,6-Tris(2-pyridyl)-s-triazine (TPTZ) were purchased from Shanghai Yuanye Bio-technology Co., Ltd. (Shanghai, China). Fluorescein was obtained from Xilong Scientific Co., Ltd. (Guangdong, China). High-performance liquid chromatography grade methanol was provided by Boda Chemical Co., Ltd. (Tianjin, China). Kanamycin sulfate was obtained from Solarbio Science and Technology Co., Ltd. (Beijing, China). The other reagents used in this study were of analytical grade and purchased from Xi’an Sanpu Chemical Reagent Co., Ltd. (Xi’an, China).

### 3.4. Determination of the Total Phenolic Content

The total phenolic content (TPC) was determined using a modified version of the Folin–Ciocalteu colorimetric method [37]. In a test tube, 0.1 mL of the solvent extract and 0.5 mL of the Folin–Ciocalteu reagent (0.2 N) were combined. After a 5 min incubation period, the mixture was neutralized with 0.5 mL of 7.5% Na_2_CO_3_. After a 1.5 h reaction, the absorbance of the mixture was measured at 765 nm using a UV–VIS spectrophotometer. The results were expressed as grams of gallic acid equivalent of (GE) per 100 g dry weight (DW) of the sample (g GE/100 g DW). The regression curves were plotted with different concentrations of gallic acid standard solutions (Supplementary Figure S3 and Table S8).

### 3.5. Determination of the Total Flavonoid Content

Total flavonoid content (TFC) was determined according to Daniela Sumczynski [38], with slight modifications. The following reagents were added to 0.5 mL of the solvent extract: 0.15 mL of 10% Al(NO_3_)_3_ and 0.15 mL of 5% NaNO_2_. After a 5 min reaction, 2 mL of a 4% NaOH was added and mixed well. Afterwards, the mixture was left for 5 min. The absorbance of the mixture was measured at 506 nm. The results are expressed as grams of rutin equivalent (RE) per 100 g of sample (g RE/100 g DW). Regression curves were plotted with different concentrations of rutin standard solutions (Supplementary Figure S4 and Table S9).

### 3.6. LC-QTOF-MS/MS Analysis

An ExionLC™ AC liquid chromatography mass spectrometer (AB SCIEX, Framingham, MA, USA) equipped with a InertSustain AQ-C18 column (4.6 mm × 150 mm × 5 μm) was used at a flow rate of 1 mL/min, a 0.1% formic acid in water (A) and methanol (B) as the mobile phase. The column temperature was maintained at 40 °C with an injection volume of 1 μL. The analytical run was set at 20 min as follows: 0–2 min, 5% B; 2–3 min, to 30% B; 3–13 min, to 85% B; 13–17 min, to 95% B; 17–18 min, 5% B; and a 2 min post-run at 5% B.

The QTOF/MS instrument was operated in negative electrospray ionization mode using the following parameters: ion source gas temperature 600 °C. Mass spectra ranged from 40 amu to 1500 amu with an ion-spray voltage of 4.5 kV; the rates of curtain, nebulizer, and heater gases were at 35 mL/min, 60 mL/min, and 40 mL/min, respectively. The ion source temperature was set at 600 °C. Data were analyzed using the Analyst 1.7 and MultiQuant 3.0.2 software [39].

### 3.7. Assessment of Antioxidant Activity with ABTS^+•^, DPPH^•^, FRAP, and ORAC Assays

ABTS^+•^ radical scavenging activity was assessed using the method as described [40], with some modifications. Equal volumes of 7.4 mM ABTS and 2.6 mM K_2_S_2_O_8_ were combined. After a 15 h equilibration period in the dark, the solution was diluted with ethanol to obtain an absorbance of 0.7 at 734 nm. Afterwards, 0.2 mL of the solvent extract was added to the diluted ABTS^+•^ solution and incubated for 6 min. Absorbance was measured at 734 nm.

DPPH radical (DPPH^•^) scavenging activity was determined as described [41], with slight modifications. In an Eppendorf tube, 0.2 mL of the solvent extract and 0.2 mL of DPPH^•^ solution were combined. After incubation for 45 min, absorbance was measured at 517 nm.

The ferric-reducing antioxidant power (FRAP) assay was performed according to the method described previously [42], with some modifications. The following reagents were combined: 2.5 mL of 10 mM TPTZ solution in 40 mM HCl, 2.5 mL of 20 mM ferric chloride, and 25 mL of 0.3 M acetate buffer. The FRAP solution was heated to 37 °C, and then 100 μL of the solvent extract was added to it. After incubation for 30 min, absorbance was measured at 593 nm.

The oxygen radical absorbance capacity (ORAC) assay is based on the scavenging of peroxyl radicals generated by 2,2′-Azobis (2-amidinopropane) dihydrochloride (AAPH); it was performed according to a previously described method [43,44,45]. In a microplate, 20 μL of the leaf extract and 20 μL of fluorescein (70 nM final concentration) were mixed and preincubated for 5 min at 37 °C. Afterwards, 150 μL of the APPH solution (12 mmol/L in PBS buffer solution) was added, and fluorescence was recorded every 2 min for 120 min at excitation and emission wavelengths of 485 and 538 nm, respectively. A blank sample containing an equal volume of methanol was prepared and analyzed.

Trolox was used as the standard for the calibration curve [46], and all results are expressed as l g of Trolox equivalents (TE) per 100 g of sample (g TE/100 g DW).

### 3.8. Assessment of Antibacterial Activity through Agar Diffusion Method

The eight bacteria which are food-borne and skin pathogens selected for this study were purchased from the BeNa Culture Collection (Supplementary Table S1). They were used to evaluate the antimicrobial activities of the leaf extracts of the nine wild tree peonies. *Streptococcus hemolytis-β* was grown in a Luria–Bertani (LB) medium [47], the other bacteria were grown on a nutrient agar (NA) medium [48]. Kanamycin (20 mg/cup) acted as the positive control, while methanol acted as the negative control. Four Gram-negative (*Escherichia coli, Salmonella enterica, Proteus vulgaris*, and *Pseudomonas aeruginosa*) and four Gram-positive bacteria (*Streptococcus hemolytis-β*, *Listeria monocytogenes*, *Propionibacterium acnes*, and *Staphylococcus aureus*) with a 1.0 × 10^8^ colony forming unit (CFU/mL) were used (CFU/mL) [35].

Minimum inhibitory concentrations (MICs) were determined according to a modified version of the method described previously [49,50]. In each microtiter well of a 96-well Elisa plate, 0.1 mL of inocula suspension and 0.1 mL of the solvent extract were combined. Dilution was performed using a standard broth microdilution method. The plate was incubated for 24 h at 37 °C. Afterwards, fluorescence was measured at 620 nm to determine the condition of microbial growth.

The Oxford cup technique was performed as previously described [51,52,53], with slight modifications. The bacteria were inoculated onto LB and NA plates, placed in five sterilized Oxford cups, and pressed lightly. Three cups were prepared corresponding to three replicates and each cup was dispensed with 0.2 mL of the solvent extract. The other two cups correspond to the control solution. The cups were incubated for 24 h at 37 °C; then, the diameters of bacterial growth inhibition zones (mm) around the cups were measured [48,54].

### 3.9. Statistical Analysis

Bioactive components, TPC and TFC, antioxidant and antibacterial activity were analyzed thrice [55]. All significance analyses were performed using the SPSS software (version 21.0 for Windows). Standardized variables with Ward linkage and squared Euclidean distance were used to perform hierarchical cluster analysis (HCA), and a correlation matrix was used to perform principal component analysis (PCA) and orthogonal partial least squares–discriminant analysis (OPLS-DA). Quantitative data of targeted compounds were imported into SIMCA 14.1 (Umetrics AB, Umea, Sweden) to generate the PCA model, HCA model, and OPLS-DA model to overview the relationship of samples. Correlation analyses were performed using the OmicStudio tool. Heat maps were plotted using the Origin software (version 2019b for Windows) [56].

## 4. Conclusions

In this study, we determined the bioactive components and bioactivities, including the antioxidant activities and antibacterial activities, of the newly grown leaves of nine WTPS. A total of 276 bioactive components were identified through non-targeted metabolomics; more than 80% of the 276 metabolites identified were terpenoids and flavonoids. A total of 42 differential metabolites were quantitatively determined. The main differential metabolites were Paeoniflorin (24.42 to 233.97 mg/mL), Luteoloside (1 to 219.94 mg/mL), Hyperin (1.34 to 103.12 mg/mL), Apigenin-7-glucoside (0.16 to 103.29 mg/mL), Rhoifolin (0.08 to 57.73 mg/mL), and Cantharidin (0 to 76.14 mg/mL). Such a high terpenoid and flavonoid content of the leaf extracts render them as species with strong antibacterial capacities, and most of the bacteria tested showed greater sensitivity to the leaf extracts derived from the members of subsection *Vaginatae* than those of subsection *Delavayanae*. Among the nine WTPS, *P. lutea* exhibited the strongest antioxidant capacity, followed by *P. jishanensis* and *P. ostii*; their strong antioxidant activity is attributed to their high TPC (9.68 to 31.18 g GE/100 g DW) and TFC (5.70 to 15.16 g RE/100 g DW). Therefore, the newly grown leaves of wild tree peonies may serve as sources of antioxidants and antimicrobials. Our results provided a theoretical basis for the in-depth application of tree peony leaves for food, cosmetic, medical, and pharmaceutical use. It is worth noting that before the widespread promotion and application of tree peony leaves as tea, food additives or medicine, we need to conduct further detailed investigations and studies on their diverse activities and possible toxicity in vivo.

## Data Availability

All relevant data are included in the manuscript and its supporting materials; further inquiries can be directed to the corresponding authors.

## Supplementary Materials

The following supporting information can be downloaded at: https://www.mdpi.com/article/10.3390/ijms241310609/s1.
