# Supplementary material for: Comprehensive Metabolite Profile Uncovers the Bioactive Components, Antioxidant and Antibacterial Activities in Wild Tree Peony Leaves
Source: Int J Mol Sci. 2023 Jun 25;24(13):10609. doi: 10.3390/ijms241310609 (PMC10342129; doi:10.3390/ijms241310609)

Supplementary Material

Supplementary Tables

Supplementary Table S1. Bacteria strain standard codes.

Latin names	bacteria codes
Staphylococcus aureus	BNCC 310011
Streptococcus hemolytis-β	BNCC 102660
Propionibacterium acnes	BNCC 336649
Listeria monocytogenes	BNCC 185986
Pseudomonas aeruginosa	BNCC 337940
Escherichia coli	BNCC 269342
Salmonella enterica subsp. *enterica*	BNCC 333565
Proteus vulgaris	BNCC 337267

Supplementary Table S2. The TPC and TFC of the newly grown leaves of nine wild tree peony species.

	TPC(g GE/100 g DW)	TFC(g RE/100 g DW)
P. jishanensis	15.83±0.79^{c}	10.85±0.78^{b}
P. qiui	11.39±0.53d^{e}	13.73±1.17^{a}
P. decomposita	9.68±0.52^{e}	8.51±0.22^{c}
P. ostii	19.71±0.21^{b}	15.16±1.29^{a}
P. rockii	15.56±1.78^{c}	14.28±0.66^{a}
P. lutea	31.18±1.30^{a}	9.08±0.03^{c}
P. delavayi	15.987±0.21^{c}	6.63±0.32^{d}
P. ludlowii	12.82±0.20^{d}	5.70±1.05^{d}
P. potaninii	20.14±0.31^{b}	12.02±0.76^{b}

Data are presented as mean ± SD (n = 3). Different superscript letters in each column indicate significant differences ($P < 0.05$).

Supplementary Table S3. Identified metabolites of newly grown leaves of nine wild tree peony species.

NO.	RT	Compound	Formula	Relative content%								
				P. jishanensis	*P. qiui*	*P. decomposita*	*P.ostii*	*P.rockii*	*P.lutea*	*P. delavayi*	*P. ludlowii*	*P. potaninii*
		**Flavonoids (27)**		33.68	23.30	49.53	82.64	31.92	38.10	42.37	22.20	25.28
1	7.4	Luteolin-7-O-β-D-glucoside	C21H20O11	5.23	6.38	13.68	25.98	15.08	2.71	13.66	7.84	10.48
2	8.1	Apigenin-7-glucoside	C21H20O10	16.04	11.00	13.64	24.01	0.31	8.39	0.06	0.08	8.02
3	7.5	Hyperin	C21H20O12	1.51	0.69	3.63	11.70	11.97	3.51	10.25	7.87	1.53
4	8.1	Rhoifolin	C27H30O14	6.15	2.36	9.85	6.76	0.11	14.34	0.08	0.04	1.59
5	10	Apigenin	C15H10O5	0.10	0.07	0.11	0.60	tr	1.41	tr	tr	0.37
6	7.3	naringenin	C15H12O5	0.04	0.03	0.02	0.05	0.01	0.09	0.07	0.04	0.01
7	9.6	Luteolin	C15H10O6	0.14	0.15	0.49	1.70	0.79	0.84	2.17	0.60	0.94

8	8.2	Daidzin	C21H20O9	0.91	1.15	2.82	4.29	0.05	0.07	0.08	0.02	0.26
9	5.9	Kaempferol-3-gentiobioside	C27H30O16	0.52	0.16	1.26	2.38	2.04	1.19	nd	1.17	nd
10	10	Isorhamnetin	C16H12O7	0.03	0.04	0.09	0.44	0.62	0.13	0.71	0.37	0.06
11	7.4	Glucosylvitexin	C27H30O15	1.97	0.56	0.96	1.21	nd	1.09	0.05	nd	0.68
12	7.9	Isoschaftoside	C26H28O14	0.56	0.21	0.97	0.46	nd	1.25	1.86	nd	0.16
13	9.1	Quercetin dihydrate	C15H10O7	0.03	0.05	0.08	0.49	0.91	0.40	2.27	0.56	0.12
14	9.6	cantharidin	C10H12O4	nd	nd	0.02	0.13	nd	2.49	7.54	3.56	0.88
15	8.2	spinosin	C28H32O15	0.34	0.45	1.82	1.75	nd	0.14	nd	nd	0.10
16	8	eriodictyol	C15H12O6	nd	tr	tr	0.48	0.01	0.01	nd	0.01	nd
17	9.2	dihydroquercetin	C15H12O7	nd	nd	0.07	0.08	nd	0.02	nd	nd	0.04

18	11	Hydroxygenkwanin	C16H12O6	tr	0.01	0.03	0.10	tr	0.01	nd	nd	tr
19	8.2	Dihydromyricetin	C15H12O8	tr	tr	nd	0.03	tr	nd	tr	tr	tr
20	6.8	Rutin	C27H30O16	nd	nd	nd	nd	nd	nd	3.55	nd	nd
21	5	Plantagoside	C21H22O12	0.10	nd	nd	nd	nd	nd	nd	nd	0.07
22	13	Tiliroside	C30H26O13	nd	nd	nd	nd	nd	nd	nd	0.03	nd
23	11	Diosmetin	C16H12O6	nd	nd	nd	nd	nd	0.01	nd	nd	nd
24	7.4	Naringin	C27H32O14	nd	nd	nd	nd	tr	nd	nd	nd	nd
25	7.6	Myricetin	C15H10O8	nd	nd	nd	nd	nd	nd	tr	nd	nd
26	17	Pectolinarin	C29H34O15	nd	nd	nd	nd	nd	nd	tr	nd	nd
27	8.8	Chrysin	C15H10O4	nd	tr	nd	nd	nd	nd	nd	nd	nd
		**Terpenoids (49)**		63.80	73.62	46.66	11.21	64.65	55.88	37.96	69.93	70.01

28	6.2	Paeoniflorin	C23H28O11	62.91	73.10	46.51	10.74	64.43	55.76	37.73	69.37	69.86
29	16	Sclareol Glycol	C16H30O2	0.05	0.04	0.05	0.09	0.05	0.04	0.12	0.05	0.04
30	9.9	Benzoylpaeoniflorin	C30H32O12	0.61	0.41	nd	nd	nd	nd	nd	nd	nd
31	5.5	Loganin	C17H26O10	nd	tr	nd	0.01	0.02	0.03	0.06	0.25	nd
32	5.4	Bilobalide	C15H18O8	0.03	0.02	0.01	0.06	0.02	nd	nd	0.12	0.03
33	5	Loganin	C17H26O10	0.17	nd	tr	0.07	0.02	nd	nd	nd	nd
34	15	Sclareolide	C16H26O2	nd	tr	tr	0.03	0.03	nd	nd	0.05	tr
35	17	Curcumol	C15H24O2	0.01	tr	tr	0.01	0.01	tr	0.02	nd	nd
36	7.2	Toosendanin	C30H38O11	tr	0.01	nd	0.03	0.02	nd	nd	nd	nd
37	15	Hederagenin	C30H48O4	tr	nd	tr	tr	0.02	tr	tr	nd	tr
38	16	Betulinic acid	C30H48O3	tr	tr	0.01	nd	tr	nd	nd	nd	nd

39	0	Genipin	C11H14O5	nd	tr	tr	tr	tr	nd	0.02	nd	nd
40	7.2	Toosendanin	C30H38O11	nd	nd	nd	nd	nd	tr	0.01	nd	tr
41	7	Dihydroartemisinin	C15H24O5	tr	tr	tr	tr	tr	nd	nd	tr	nd
42	18	Notoginsenoside Fe	C47H80O17	tr	tr	nd	nd	nd	nd	nd	nd	tr
43	16	Kaurenoic acid	C20H30O2	nd	tr	nd	nd	nd	tr	nd	tr	nd
44	13	Carvacrol	C10H14O	tr	tr	tr	tr	nd	nd	nd	nd	tr
45	16	Tussilagone	C23H34O5	tr	nd	nd	tr	nd	nd	nd	tr	nd
46	9.3	Lappaconite Hydrobromide	C32H44N2O8	nd	tr	nd	tr	nd	nd	nd	nd	nd
47	12	Linderane	C15H16O4	nd	tr	nd	tr	tr	nd	nd	nd	nd
48	9.3	Saikosaponin C	C48H78O17	tr	tr	nd	tr	nd	nd	nd	tr	nd
49	15	Costunolide	C15H20O2	tr	tr	tr	tr	nd	nd	nd	nd	tr

50	6.3	6-deoxycatalpol	$C_{15}H_{22}O_9$	nd	nd	nd	nd	nd	nd	nd	0.09	nd
51	7.9	pseudolaric acid B	$C_{23}H_{28}O_8$	nd	nd	nd	nd	nd	nd	nd	nd	0.06
52	16	Benzoylhypacoitine	$C_{31}H_{43}NO_9$	nd	nd	nd	0.11	nd	nd	nd	nd	nd
53	17	Andrographolide	$C_{20}H_{30}O_5$	nd	nd	0.03	nd	nd	nd	nd	nd	nd
54	7.3	Ginkgolide C	$C_{20}H_{24}O_{11}$	nd	nd	nd	nd	0.01	nd	nd	nd	nd
55	18	Nardosinone	$C_{15}H_{22}O_3$	nd	nd	nd	nd	nd	nd	nd	nd	0.01
56	14	Asiatic acid	$C_{30}H_{48}O_5$	nd	nd	nd	nd	nd	tr	nd	nd	tr
57	11	Andrographolide	$C_{20}H_{30}O_5$	nd	nd	nd	tr	tr	nd	nd	nd	nd
58	16	Glycyrrhetinic acid	$C_{30}H_{46}O_4$	nd	tr	nd	nd	nd	nd	nd	nd	nd
59	11	Tenuifolin	$C_{36}H_{56}O_{12}$	nd	nd	nd	nd	tr	nd	nd	tr	nd

60	6.1	Neonuezhenide	C31H42O18	nd	nd	nd	nd	nd	tr	nd	nd	nd
61	7.7	Ginkgolide A	C20H24O9	nd	nd	nd	nd	nd	tr	nd	nd	nd
62	5.7	Artemisinin	C15H22O5	nd	nd	tr	nd	nd	tr	nd	nd	nd
63	5.4	Geniposide	C17H24O10	nd	tr	nd	tr	nd	nd	nd	nd	nd
64	15	Atractylenolide Ⅲ	C15H20O3	nd	nd	tr	nd	nd	nd	nd	tr	tr
65	9.4	Paclitaxel	C47H51NO14	nd	nd	nd	nd	nd	tr	nd	nd	nd
66	16	Betulinic acid	C30H48O3	nd	nd	nd	nd	nd	tr	nd	nd	nd
67	14	Picfeltarraenin IB	C42H64O14	nd	nd	tr	nd	nd	nd	nd	nd	nd
68	15	Raddeanin A	C47H76O16	nd	nd	tr	nd	nd	nd	nd	nd	nd
69	6.1	Atractylenolide I	C15H18O2	nd	nd	nd	tr	nd	nd	nd	nd	nd

70	11	Pedunculoside	C36H58O10	nd	nd	nd	nd	tr	nd	nd	nd	nd
71	5.9	Solanesol	C45H74O	nd	tr	nd	nd	nd	nd	nd	nd	nd
72	16	Tenacissoside H	C42H66O14	nd	nd	nd	nd	nd	nd	nd	nd	tr
73	18	Shionone	C30H50O	nd	tr	nd	nd	nd	nd	nd	nd	nd
74	14	Phytolaccagenin	C31H48O7	nd	tr	nd	nd	nd	nd	nd	nd	nd
75	7.8	Pulegone	C10H16O	nd	tr	nd	nd	nd	nd	nd	nd	nd
76	8.9	Limonin	C26H30O8	nd	nd	nd	tr	nd	nd	nd	nd	nd
		**Quinones (7)**		tr	tr	tr	tr	0.01	0.01	0.02	tr	tr
77	9.9	Aurantio-obtusin	C17H14O7	nd	nd	nd	nd	tr	tr	nd	nd	tr
78	8.2	Purpurin	C14H8O5	nd	nd	nd	nd	tr	tr	0.01	tr	nd
79	5.2	Plumbagin	C11H8O3	nd	tr	tr	nd	tr	tr	tr	nd	tr
80	7.4	Rhein	C15H8O6	nd	nd	tr	nd	nd	nd	nd	nd	nd

81	18	Shikonin	C16H16O5	nd	nd	nd	nd	tr	nd	nd	nd	tr
82	10	1,8-Dihydroxyanthraquinone	C14H8O4	nd	tr	nd	nd	nd	nd	nd	nd	nd
83	18	Dictamnine	C12H9NO2	nd	nd	nd	nd	nd	nd	nd	nd	tr
		**Lipids (11)**		0.23	0.61	0.93	1.60	1.04	1.45	3.69	1.90	0.64
84	16	Linoleic acid	C18H32O2	0.22	0.32	0.54	1.07	0.64	1.29	3.66	1.43	0.63
85	8	Polygalic acid	C29H44O6	nd	0.28	0.34	0.52	0.37	nd	nd	nd	nd
86	5.5	10-Hydroxy-2-decenoic acid	C10H18O3	tr	tr	0.01	nd	0.03	0.10	nd	nd	nd
87	7	Pogostone	C12H16O4	nd	tr	tr	nd	tr	nd	0.01	nd	tr
88	17	Methyl linoleate	C19H34O2	nd	nd	nd	tr	nd	tr	tr	tr	nd
89	16	Ganoderic acid H	C32H44O9	nd	nd	nd	nd	nd	nd	nd	0.36	nd
90	18	Tigogenin	C27H44O3	nd	nd	0.05	nd	nd	nd	nd	nd	nd

91	15	Gracillin	C45H72O17	tr	nd	nd	nd	nd	nd	nd	nd	nd
92	18	Sodium glycodeoxycholate	C26H42NNaO5	tr	nd	nd	nd	nd	nd	nd	nd	nd
93	17	10-Gingerol	C21H34O4	nd	nd	nd	tr	nd	nd	nd	nd	nd
94	18	Anise oil	C10H12O	tr	nd	tr	tr	nd	0.05	0.01	0.11	tr
		**Hydrocarbons (10)**		0.39	0.51	0.68	0.12	0.34	0.99	6.82	0.42	2.12
95	6.2	Paeonol	C9H10O3	nd	0.03	nd	0.10	0.12	0.70	0.16	0.05	1.21
96	10	Heteroclitin D	C27H30O8	0.39	0.45	0.67	nd	0.22	0.29	nd	0.35	0.89
97	6	Pinoresinol Diglucoside	C32H42O16	nd	nd	nd	nd	tr	nd	0.06	0.02	0.02
98	8.5	Lobetyolin	C20H28O8	tr	tr	0.01	0.02	nd	nd	nd	nd	nd
99	5.6	Schisandrin C	C22H24O6	nd	0.01	nd	nd	nd	nd	nd	nd	nd
100	7.3	Kaempferol-3-o-rutinoside	C27H30O15	nd	nd	nd	nd	nd	nd	6.58	nd	nd

101	13	Erianin	C18H22O5	nd	nd	nd	nd	nd	nd	0.01	nd	nd
102	9.6	Curcumin	C21H20O6	nd	nd	nd	nd	nd	nd	tr	nd	nd
103	1.2	Cryptotanshinone	C19H20O3	nd	nd	nd	nd	nd	nd	nd	nd	nd
104	7.7	Resveratrol	C14H12O3	nd	0.02	nd	tr	nd	tr	nd	tr	nd
		**Glycosides (48)**		0.24	0.53	0.98	1.51	0.26	1.09	3.32	3.66	0.55
105	6.2	Curculigoside	C22H26O11	0.10	0.22	0.40	0.58	0.10	0.17	0.08	0.14	0.28
106	5.9	Complanatoside	C28H32O16	nd	0.02	0.39	0.05	0.02	0.17	0.97	nd	0.03
107	5.5	Sesamoside	C17H24O12	nd	nd	nd	nd	nd	0.20	0.28	0.49	0.08
108	5.3	sweroside	C16H22O9	nd	nd	nd	nd	nd	0.21	nd	0.18	tr
109	9.5	Harpagosid	C24H30O11	nd	0.05	0.04	nd	0.03	0.08	0.08	0.10	0.03
110	7.7	Phloridzin	C21H24O10	0.03	0.13	nd	0.08	0.03	nd	nd	nd	nd
111	5.7	Gentiopicrin	C16H20O9	nd	nd	nd	nd	nd	0.06	0.16	0.10	0.03

112	5.4	Syringin	C17H24O9	tr	0.02	tr	0.24	tr	tr	0.04	0.03	0.04
113	1.1	Dipsacoside B	C17H26O11	0.04	nd	0.06	0.07	0.04	nd	nd	nd	nd
114	7.1	(-)-Pinoresinol glucoside	C26H32O11	0.02	0.01	0.01	0.04	0.03	nd	0.03	2.34	0.01
115	16	Astragaloside Ⅳ	C41H68O14	0.02	0.02	0.01	0.03	tr	nd	nd	tr	tr
116	5	10-deacetylasperulosidic acid	C16H22O11	nd	nd	nd	0.02	nd	0.03	nd	0.04	nd
117	6.4	Polydatin	C20H22O8	nd	0.03	0.01	nd	nd	nd	nd	nd	nd
118	6.8	Wogonin 7-O-glucuronide	C22H20O11	nd	tr	tr	nd	tr	nd	tr	tr	0.01
119	7.6	Tracheloside	C27H34O12	nd	nd	tr	nd	nd	nd	nd	nd	0.02
120	16	Harpagide	C15H24O10	nd	nd	tr	tr	tr	nd	tr	tr	tr
121	1.1	Salicin	C13H18O7	tr	tr	tr	nd	tr	nd	nd	nd	nd

122	17	Bufotaline	C26H36O6	nd	nd	tr	nd	nd	nd	tr	tr	nd
123	7.1	Mulberroside A	C26H32O14	nd	nd	0.01	0.33	nd	nd	1.59	nd	nd
124	7.5	neoeriocitrin	C27H32O15	nd	nd	nd	nd	nd	0.13	nd	0.02	nd
125	9.8	Etoposide	C29H32O13	nd	nd	nd	nd	nd	nd	nd	0.10	nd
126	6.3	Glucosylvitexin	C27H30O15	nd	nd	nd	nd	nd	nd	nd	0.08	nd
127	6.2	Prim-O-glucosylcimifugin	C22H28O11	0.02	nd	nd	0.03	nd	nd	nd	nd	nd
128	6.1	Arbutin	C12H16O7	nd	0.02	nd	nd	nd	nd	nd	nd	nd
129	8.7	calycosin-7-o-glucoside	C22H22O10	tr	nd	nd	nd	nd	tr	0.02	nd	tr
130	6.1	Vaccarin	C32H38O19	nd	nd	nd	nd	nd	0.02	nd	nd	nd
131	5.8	Catalpol	C15H22O10	nd	nd	nd	nd	nd	nd	0.02	tr	nd

132	6.2	Methylhesperidin	C29H36O15	tr	nd	nd	0.02	nd	nd	nd	nd	nd
133	6.3	Geniposidic acid	C16H22O10	nd	nd	nd	nd	nd	nd	0.02	nd	nd
134	9.9	Icarrin	C33H40O15	nd	tr	nd	nd	nd	nd	nd	tr	nd
135	5.5	Aucubin	C15H22O9	nd	nd	nd	nd	nd	nd	0.02	nd	nd
136	16	Ziyuglycoside I	C41H66O13	tr	tr	nd	nd	nd	nd	nd	nd	nd
137	15	Liriope muscari baily saponins C	C44H70O17	nd	nd	tr	nd	nd	nd	nd	tr	nd
138	5	Nodakenin	C20H24O9	nd	tr	tr	nd	nd	nd	nd	nd	nd
139	6.3	Asperuloside	C18H22O11	nd	nd	nd	0.02	nd	nd	nd	nd	nd
140	17	Pseuoginsenoside F11	C42H72O14	nd	tr	nd	nd	nd	tr	nd	nd	tr
141	19	Pseudoginsenoside-RT5	C36H62O10	nd	nd	nd	nd	nd	tr	nd	nd	nd

142	6.2	Swertimarin	C16H22O10	nd	nd	nd	tr	nd	nd	nd	nd	nd
143	17	Notoginsenoside Ft1	C47H80O17	nd	nd	nd	nd	nd	nd	tr	nd	nd
144	8.3	Secoisolariciresinol diglucoside	C32H46O16	nd	nd	nd	nd	nd	nd	tr	nd	nd
145	18	Ophiogenin 3-O-α-L-rhamnopyranosyl-(1→2)-β-D-glucopyranoside	C39H62O14	nd	nd	nd	nd	tr	nd	nd	nd	nd
146	6.3	Orcinol glucosid	C13H18O7	nd	nd	nd	tr	nd	nd	nd	nd	nd
147	14	Neoandrographolide	C26H40O8	nd	tr	nd	nd	nd	nd	nd	nd	nd
148	15	Asperosaponin Ⅵ	C47H76O18	tr	nd	nd	nd	nd	nd	nd	nd	nd
149	8.4	Phillyrin\Forsythin	C27H34O11	nd	nd	nd	nd	tr	nd	nd	nd	nd

150	18	Saikosaponin B2	C42H68O13	nd	nd	nd	nd	nd	nd	nd	tr	nd
151	19	α-hederin	C41H66O12	nd	nd	nd	nd	nd	nd	nd	tr	nd
152	0.1	Salidroside	C14H20O7	nd	nd	nd	nd	nd	nd	tr	nd	nd
		**Carboxylic Acids (21)**		1.43	0.93	0.73	1.82	1.11	1.72	3.26	1.10	0.62
153	1.1	quinic acid	C7H12O6	1.26	0.59	0.47	1.38	0.54	0.78	1.16	0.12	0.30
154	1.1	Gallic acid	C7H6O5	0.07	0.03	0.03	0.11	0.09	0.19	0.49	0.14	0.07
155	1.2	Citric acid	C6H8O7	nd	0.11	0.14	0.26	0.26	0.38	0.25	0.36	0.09
156	1.2	malic acid	C4H6O5	nd	0.10	nd	nd	0.13	0.32	0.56	0.43	0.12
157	5.2	protocatechuic acid	C7H6O4	0.03	0.04	0.05	nd	0.08	0.01	0.09	0.03	nd
158	18	Ethyl ferulate	C12H14O4	tr	tr	0.01	0.02	tr	tr	0.01	tr	nd
159	0.1	Hydroxycitric acid	C6H8O8	0.01	nd	nd	nd	0.01	tr	tr	nd	tr
160	6.5	Danshensu	C9H10O5	nd	nd	nd	tr	tr	0.01	nd	nd	nd

161	17	Euphorbiast eroid	C32H40O8	tr	nd	nd	nd	nd	nd	tr	tr	nd
162	6.7	p-Coumaric acid	C9H8O3	nd	nd	nd	nd	nd	0.01	0.37	nd	nd
163	9.6	Vanillic acid	C8H8O4	nd	nd	nd	nd	nd	nd	0.26	nd	nd
164	7.8	salicylic acid	C7H6O3	nd	nd	nd	nd	nd	nd	nd	nd	0.04
165	8.4	3,4-Dimethoxyb enzoic acid	C9H10O4	nd	nd	nd	nd	nd	nd	0.06	nd	nd
166	7.7	p-Anisic acid	C8H8O3	0.02	nd	nd	nd	nd	nd	nd	nd	nd
167	9.6	2-Methoxycin namic acid	C10H10O3	nd	nd	nd	0.04	nd	nd	nd	nd	nd
168	8.6	Cichoric acid	C22H18O1 2	tr	nd	nd	nd	nd	nd	nd	tr	nd
169	6.1	3,6’ — Disinapoyl sucrose	C34H42O1 9	nd	nd	nd	nd	nd	nd	nd	tr	nd
170	0	Ferulic Acid	C10H10O4	tr	nd	tr	nd	nd	nd	nd	nd	nd

171	12	Chlorogenic acid	$C_{16}H_{18}O_9$	nd	tr	tr	nd	nd	nd	nd	nd	nd
172	6.3	Phenprobamate	$C_9H_{11}NO_2$	nd	tr	nd	nd	nd	nd	nd	nd	tr
173	6.1	Epicatechin gallate	$C_{22}H_{18}O_{10}$	0.02	0.05	0.02	nd	nd	nd	nd	nd	nd
		**Benzopyrans (27)**		0.03	0.12	0.02	0.09	0.11	0.14	0.14	0.14	0.05
174	7.8	Ellagic Acid	$C_{14}H_6O_8$	nd	0.10	nd	0.04	0.06	nd	nd	nd	0.04
175	9.4	Hamaudol Glycoside	$C_{21}H_{26}O_{10}$	tr	nd	nd	nd	nd	0.08	0.11	0.09	nd
176	5.4	Isopimpinellin	$C_{13}H_{10}O_5$	nd	nd	nd	nd	nd	0.02	0.01	0.02	nd
177	19	Liquiritigenin	$C_{15}H_{12}O_4$	nd	nd	tr	nd	0.01	tr	0.01	0.03	nd
178	5.1	3-N-butyl-4,5-dihydrophthalide	$C_{12}H_{16}O_2$	nd	tr	tr	tr	tr	tr	nd	nd	nd
179	7	Cimifugin	$C_{16}H_{18}O_6$	tr	nd	tr	0.03	nd	nd	nd	nd	nd

180	5.5	Baicalin	C21H18O11	0.01	nd	nd	nd	nd	nd	nd	nd	nd
181	7	6-hydroxy-7,8-dimethoxycoumarin	C11H10O5	nd	nd	nd	tr	tr	tr	tr	nd	tr
182	5	Epicatechin	C15H14O6	nd	tr	nd	tr	nd	tr	nd	nd	nd
183	6.8	Marmesin	C14H14O4	nd	nd	nd	tr	tr	tr	nd	tr	nd
184	9.4	Wedelolactone	C16H10O7	tr	tr	nd	nd	tr	tr	nd	nd	nd
185	0	6,7-Dimethoxycoumarin	C11H10O4	tr	tr	tr	tr	tr	nd	nd	nd	nd
186	5.8	Fraxin	C16H18O10	nd	tr	tr	tr	nd	nd	nd	nd	nd
187	5.7	4-Hydroxycoumarin	C9H6O3	tr	tr	nd	nd	tr	nd	nd	nd	nd
188	12	Pectolinarigenin	C17H14O6	nd	tr	nd	nd	tr	nd	nd	nd	nd
189	5.3	Bergeninum	C14H16O9	nd	nd	nd	nd	0.01	tr	nd	nd	nd

190	6	Fraxin	C16H18O10	nd	nd	nd	nd	nd	nd	nd	nd	0.01
191	11	Oxypeucedanin	C16H14O5	nd	nd	nd	nd	nd	0.01	nd	nd	nd
192	12	6-Methylcoumarin	C10H8O2	nd	nd	nd	nd	tr	tr	nd	nd	nd
193	1.1	Catechin	C15H14O6	tr	nd	nd	nd	tr	nd	nd	nd	nd
194	5.2	Fraxetin	C10H8O5	nd	nd	tr	nd	tr	nd	nd	nd	tr
195	6.4	Osthole	C15H16O3	nd	nd	tr	tr	tr	nd	tr	tr	nd
196	6.4	Esculetin	C9H6O4	nd	tr	nd	nd	nd	nd	nd	nd	tr
197	5.5	Cistanoside D	C31H40O15	nd	nd	nd	nd	nd	nd	nd	nd	tr
198	7.6	fraxinellone	C14H16O3	nd	nd	nd	tr	nd	tr	nd	nd	nd
199	11	Brazilin	C16H14O5	nd	tr	nd	nd	nd	nd	nd	nd	nd
200	7.6	Imperatorin	C16H14O4	nd	nd	nd	nd	nd	nd	nd	tr	nd
		**Amino Acids (10)**		tr	0.02	0.04	0.03	0.06	0.03	0.10	0.07	0.38

201	1	Aspartic acid	C4H7NO4	tr	nd	tr	tr	tr	tr	0.01	tr	0.26
202	1.2	Proline	C5H9NO2	nd	nd	nd	nd	nd	nd	tr	tr	0.10
203	1.1	Amber Acid	C4H6O4	nd	nd	tr	0.02	0.02	0.01	0.03	0.03	tr
204	1	Glutamic acid	C5H9NO4	nd	0.02	0.02	nd	0.02	tr	0.02	0.02	tr
205	4.8	L-Tryptophan	C11H12N2O2	tr	nd	tr	nd	tr	tr	0.01	0.01	tr
206	1	Serine	C3H7NO3	nd	tr	tr	tr	tr	tr	tr	tr	tr
207	1.2	Leucine	C6H13NO2	nd	tr	nd	tr	tr	nd	tr	nd	tr
208	6.7	L(+)-Arginine	C6H14N4O2	nd	nd	nd	tr	tr	nd	tr	nd	nd
209	16	Histidine	C6H9N3O2	tr	nd	tr	nd	nd	nd	nd	nd	nd
210	18	Citrulline	C6H13N3O3	nd	nd	nd	nd	nd	nd	nd	nd	tr
		**Alkaloids (15)**		0.01	0.02	0.03	tr	0.03	0.03	0.02	0.01	0.02
211	9.1	Irinotecan	C33H38N4O6	tr	tr	nd	nd	0.03	nd	nd	nd	nd

212	17	(+)-Tetrandrine	C38H42N2O6	nd	tr	nd	nd	nd	nd	0.01	tr	tr
213	16	Catharanthine	C21H24N2O2	nd	tr	nd	tr	tr	nd	nd	nd	tr
214	11	Canadine	C20H21NO4	nd	nd	nd	nd	nd	tr	tr	nd	nd
215	5.6	Topotecan	C23H23N3O5	tr	nd	nd	nd	nd	nd	nd	nd	0.01
216	16	Caffeine	C8H10N4O2	nd	nd	nd	nd	nd	0.02	nd	nd	tr
217	9.3	Reserpine	C33H40N2O9	nd	tr	0.02	nd	nd	nd	nd	nd	nd
218	0.1	Trigonelline	C7H7NO2	nd	nd	tr	nd	nd	tr	nd	nd	nd
219	8	Colchicine	C22H25NO6	nd	nd	nd	nd	nd	tr	nd	tr	nd
220	8	Gramine	C11H14N2	tr	nd	nd	nd	tr	tr	nd	nd	nd
221	10	Sophoridine	C15H24N2O	nd	nd	nd	tr	nd	nd	nd	nd	nd
222	7.8	Abrine	C12H14O2N2	nd	nd	nd	nd	nd	nd	nd	tr	nd

223	8.2	Lycorine	C16H17NO4	nd	nd	nd	nd	tr	nd	nd	nd	nd
224	10	Peimisine	C27H41NO3	nd	nd	nd	nd	nd	nd	nd	nd	tr
225	5.3	Higenamine	C16H17NO3	nd	nd	tr	nd	nd	nd	nd	nd	nd
		**Vitamins (7)**		tr	0.02	0.23	0.49	0.25	0.19	tr	0.15	tr
226	5.1	Vitamin C	C6H8O6	nd	nd	0.22	0.48	0.25	0.19	nd	0.03	nd
227	7.6	Vitamin B5	C18H32CaN2O10	nd	0.02	nd	0.01	tr	nd	nd	0.12	nd
228	16	Vitamin A acid	C20H28O2	tr	tr	tr	nd	tr	tr	nd	nd	nd
229	0.1	Arecoline hydrobromide	C8H13NO2	nd	tr	nd	tr	nd	nd	nd	tr	nd
230	0	Nicotinic acid	C6H5NO2	tr	nd	tr	nd	nd	nd	nd	nd	nd
231	11	Menadione (Vitamin K3)	C11H10O5S	nd	nd	nd	nd	tr	nd	nd	nd	nd

		Bisulfurous Acid										
232	5.1	Vitamin B6	C8H11NO3	nd	nd	nd	nd	nd	tr	nd	nd	nd
		**Phenolic acids (2)**		tr	tr	tr	tr	tr	tr	tr	tr	tr
233	6.7	Benzoic acid	C10H12O5	nd	tr	tr	tr	tr	nd	nd	nd	nd
234	6.8	Caffeic acid	C9H8O4	tr	nd	nd	nd	tr	nd	nd	nd	nd
		**Heterocyclic Compounds (6)**		tr	tr	tr	0.07	0.03	tr	2.25	tr	tr
235	15	Levistilide A	C24H28O4	tr	tr	tr	0.01	tr	nd	nd	nd	tr
236	8.2	Agarotetrol	C17H18O6	nd	nd	nd	0.06	0.02	nd	2.25	nd	nd
237	15	Gambogic acid	C38H44O8	nd	tr	nd	nd	nd	nd	nd	nd	nd
238	1.1	Pipecolinic acid	C6H11NO2	nd	nd	nd	tr	nd	nd	nd	nd	nd
239	6.8	Phellodendrine	C20H23NO4	nd	nd	nd	nd	tr	nd	nd	nd	nd

240	7	Thymine	C5H6N2O2	nd	nd	nd	nd	nd	nd	nd	nd	tr
		**Aldehydes (5)**		0.02	0.03	0.08	0.03	0.05	tr	0.02	0.02	tr
241	7.7	Isovanillin	C8H8O3	nd	0.02	0.08	nd	0.05	nd	nd	nd	nd
242	1.1	5-Hydroxymethylfurfural	C6H6O3	0.02	tr	nd	0.03	nd	nd	nd	nd	nd
243	8.4	Syringaldehyde	C9H10O4	nd	nd	nd	nd	nd	nd	nd	0.02	nd
244	7.8	Helicid	C13H16O7	nd	nd	nd	nd	nd	nd	0.02	nd	nd
245	0.1	Anisaldehyde	C8H8O2	nd	tr	nd	nd	tr	nd	nd	nd	nd
		**Ketones (3)**		0.09	0.27	0.06	0.26	0.12	0.31	tr	0.34	0.25
246	8.2	（tb-B3）7一O一methylmangiferin	C20H20O11	0.09	0.25	0.06	0.24	0.12	0.31	nd	0.33	0.25
247	9.5	Agrimol B	C37H46O12	nd	0.02	nd	0.02	nd	nd	nd	nd	nd

248	12	Raspberry ketone	C10H12O2	nd	tr	tr	tr	tr	nd	nd	0.01	nd
		**Phytosterols (2)**		nd	tr	nd	nd	tr	nd	nd	nd	nd
249	6.1	Epibrassinolide	C28H48O6	nd	tr	nd	nd	nd	nd	nd	nd	nd
250	16	Hydroxyecdysone	C27H44O7	nd	nd	nd	nd	tr	nd	nd	nd	nd
		**Others (26)**		0.05	tr	0.02	0.09	0.03	0.06	0.04	0.05	0.06
251	6.2	Hydroxytyrosol	C8H10O3	tr	nd	tr	tr	tr	nd	nd	nd	0.02
252	5.9	Tyrosol	C8H10O2	tr	nd	tr	tr	tr	nd	nd	tr	nd
253	4.8	bomyl acetate	C12H20O2	tr	tr	tr	nd	tr	tr	tr	nd	tr
254	9.3	Ligustilide	C12H14O2	nd	tr	nd	nd	nd	nd	nd	nd	nd
255	12	Isomucronulatol	C17H18O5	nd	nd	tr	0.02	nd	nd	nd	nd	nd
256	16	Liriopesides B	C39H62O12	tr	nd	tr	tr	tr	tr	nd	nd	nd

257	15	Nobiletin	C21H22O8	tr	nd	tr	nd	tr	nd	nd	nd	nd
258	9.7	Cistanoside C	C30H38O15	nd	nd	nd	nd	nd	0.04	nd	nd	0.03
259	1.1	（tb-C1）Polygalaxanthone Ⅵ	C23H26O12	0.03	nd	nd	nd	nd	nd	nd	nd	nd
260	8.3	epimeredinoside A	C31H40O15	nd	nd	nd	nd	nd	0.02	nd	0.02	nd
261	17	8-Gingerol	C19H30O4	nd	nd	nd	0.05	nd	nd	nd	nd	nd
262	4.7	α-Mangostin	C24H26O6	nd	nd	nd	nd	nd	nd	0.03	nd	nd
263	9.4	Sodium Taurocholate	C26H44NNaO7S	nd	nd	nd	0.01	tr	nd	nd	nd	nd
264	20	D-Sorbitol	C6H14O6	tr	tr	nd	nd	nd	nd	tr	nd	nd
265	9	(-)-Syringaresnol-4-O-β-D-apiofuranosyl-(1→2)-β-D-glucopyran	C33H44O17	nd	nd	nd	nd	nd	nd	nd	tr	nd

266	5.4	Drcorhodin perochlorate	C17H14O3	nd	nd	nd	nd	nd	nd	nd	tr	nd
267	7.6	Levodopa	C9H11NO4	nd	nd	nd	tr	nd	nd	nd	nd	nd
268	13	Scopolamine Hydrobromide	C17H21NO4	nd	nd	nd	nd	nd	nd	nd	tr	nd
269	14	Tauroursodeoxycholic acid	C26H45NO6S	nd	nd	nd	nd	nd	nd	nd	nd	tr
270	10	Phenobarbital	C12H12N2O3	nd	nd	nd	nd	nd	nd	nd	tr	nd
271	6.6	Atropine sulfate	C17H23NO3	tr	nd	nd	nd	nd	nd	nd	nd	nd
272	9.7	4-Hydroxytolbutamide	C12H18N2O4S	nd	nd	tr	nd	nd	nd	nd	nd	nd
273	5.3	Esculin hydrate	C15H16O9	nd	nd	nd	nd	nd	nd	nd	0.02	nd
274	11	Butylparaben	C11H14O3	nd	nd	nd	nd	0.02	nd	nd	nd	0.01

275	6.2	Oxyresveratrol	C14H12O4	nd	tr	nd	nd	nd	nd	nd	nd	nd
276	8.4	(-)-gallocatechin	C15H14O7	nd	nd	nd	nd	nd	nd	tr	nd	nd

“nd” indicates that the substance was not detected under analytical conditions used; “tr”, trace (<0.01%)

Supplementary Table S4. Relative content (%) of bioactive compounds belonging to different biochemical classes in the newly grown leaves of nine wild tree peony species.

	P. jishanensis	*P. qiui*	*P. decomposita*	*P.ostii*	*P.rockii*	*P.lutea*	*P. delavayi*	*P. ludlowii*	*P. potaninii*
Terpenoids	63.80	73.62	46.66	11.21	64.65	55.88	37.96	69.93	70.01
Flavonoids	33.68	23.30	49.53	82.64	31.92	38.10	42.37	22.20	25.28
Lipids	0.23	0.61	0.93	1.60	1.04	1.45	3.69	1.90	0.64
Hydrocarbons	0.39	0.51	0.68	0.12	0.34	0.99	6.82	0.42	2.12
Glycosides	0.24	0.53	0.98	1.51	0.26	1.09	3.32	3.66	0.55
Carboxylic Acids	1.43	0.93	0.73	1.82	1.11	1.72	3.26	1.10	0.62
Amino acids	0.01	0.02	0.04	0.03	0.06	0.03	0.10	0.07	0.38
Phenolic acids	0.00	0.00	0.00	0.01	0.00	0.00	0.00	0.00	0.00
Benzopyrans	0.03	0.12	0.02	0.09	0.11	0.14	0.14	0.14	0.05
Quinones	0.00	0.00	0.01	0.00	0.01	0.01	0.02	0.00	0.01

Alkaloids	0.01	0.02	0.03	0.01	0.03	0.03	0.02	0.01	0.02
Vitamins	0.00	0.02	0.23	0.49	0.25	0.19	0.00	0.15	0.00
Heterocyclic Compounds	0.00	0.01	0.01	0.07	0.03	0.00	2.25	0.00	0.00
Aldehydes	0.02	0.03	0.08	0.03	0.05	0.00	0.02	0.02	0.00
Ketones	0.09	0.27	0.06	0.26	0.12	0.31	0.00	0.34	0.25
Others	0.05	0.01	0.02	0.09	0.03	0.06	0.04	0.05	0.06

Supplementary Table S5. Absolute content (mg/ml) of 42 differential metabolites in newly grown leaves of nine wild tree peony species.

NO.	RT	Compound	Formula	Class	Absolute content (mg/ml								
					P. jishanensis	*P. qiui*	*P. decomposita*	*P.ostii*	*P.rockii*	*P. lutea*	*P. delavayi*	*P. ludlowii*	*P. potaninii*
1	6.24	Paeoniflorin	C23H28O11	Terpenoids	93.330 ±2.419^{e}	101.230 ±4.616^{de}	214.293 ±18.983^{a}	118.639 ±7.026^{cd}	152.795 ±2.539^{b}	219.396 ±1.967^{a}	138.909 ±3.575^{bc}	233.969 ±5.723^{a}	24.415 ±3.249^{f}
2	7.37	Luteoloside	C21H20O11	Flavonoids	20.279 ±1.219^{de}	219.940 ±7.422^{a}	57.737± 14.025^{c}	12.037± 1.646^{e}	12.348 ±0.971^{e}	32.506 ±0.752^{d}	16.523± 0.701^{de}	148.662 ±9.186^{b}	2.939± 0.246^{e}
3	7.53	Hyperin	C21H20O12	Flavonoids	16.905 ±0.435^{c}	61.136± 5.971^{b}	18.977± 1.014^{c}	1.341±0.394^{d}	3.340± 0.457^{d}	4.725± 0.128^{d}	16.322± 0.813^{c}	103.124 ±13.084^{a}	3.314± 0.372^{d}
4	5.88	Kaempferol-3-gentiobioside	C27H30O16	Flavonoids	2.924± 0.253^{bc}	11.325± 0.918^{a}	3.987±2.146^{b}	0.315±0.069^{e}	1.241± 0.041^{de}	0^{e}	2.342±0.260^{cd}	0^{e}	1.141± 0.076^{de}
5	9.1	Quercetin dihydrate	C15H10O7	Flavonoids	1.167± 0.163^{b}	1.712±0.784^{b}	0.397±0.182^{b}	0.053±0.005^{b}	0.060± 0.002^{b}	0.379± 0.020^{b}	1.153±0.101^{b}	23.287± 3.749^{a}	0.383± 0.116^{b}
6	9.6	Luteolin	C15H10O6	Flavonoids	1.042± 0.109^{c}	7.781±0.447^{b}	2.326±0.151^{c}	0.283±0.037^{c}	0.332± 0.013^{c}	2.868± 0.112^{c}	1.222±0.021^{c}	22.451± 3.409^{a}	0.870± 0.094^{c}
7	10	Isorhamnetin	C8H8O3	Flavonoids	0.820± 0.091^{bc}	1.601±0.666^{b}	0.618±0.787^{bc}	0.059±0.031^{c}	0.063± 0.001^{c}	0.176± 0.017^{c}	0.751±0.012^{bc}	6.964±1.209^{a}	0.125± 0.045^{c}
8	8.07	Apigenin-7-glucoside	C21H20O10	Flavonoids	0.437± 0.047^{e}	103.290 ±9.263^{a}	73.676± 4.831^{b}	18.031± 3.224^{d}	37.842 ±2.760^{c}	26.300 ±1.726^{d}	0.160±0.025^{e}	0.518±0.148^{e}	8.019± 0.887^{e}

9	8.21	7-O-methylmangiferin	C20H20O11	Ketones	0.167±0.005^{bc}	0.813±0.686^{b}	0.306±0.006^{abc}	0.391±0.056^{abc}	0.180±0.065^{bc}	0.836±0.107^{a}	0.656±0.009^{ab}	0^{c}	0.154±0.017^{bc}
10	8.1	Rhoifolin	C27H30O14	Flavonoids	0.133±0.042^{e}	40.571±7.332^{b}	57.736±6.212^{a}	4.755±0.486^{de}	13.468±1.889^{c}	4.858±0.718^{de}	0.083±0.004^{e}	0.819±0.084^{e}	11.082±1.389^{cd}
11	9.57	Cantharidin	C10H12O4	Flavonoids	0.149±0.015^{c}	2.271±0.938^{a}	1.187±0.144^{b}	0.585±0.014^{bc}	0.254±0.014^{c}	0.980±0.082^{bc}	0.300±0.017^{c}	0.844±0.125^{bc}	0.122±0.007^{c}
12	6.15	Curculigoside	C22H26O11	Glycosides	0.119±0.004^{b}	0.362±0.181^{b}	0.159±0.005^{b}	0.055±0.010^{b}	0.153±0.013^{b}	0.198±0.014^{b}	0.266±0.041^{b}	4.349±1.136^{a}	0.128±0.039^{b}
13	1.13	Gallic acid	C7H6O5	Carboxylic Acids	0.067±0.006^{b}	0^{d}	0.401±0.012^{a}	0.041±0.002^{c}	0^{d}	0^{d}	0^{d}	0^{d}	0^{d}
14	7.65	Isovanillin	C8H8O3	Aldehydes	0.038±0.002^{b}	0.300±0.138^{a}	0^{b}	0.365±0.052^{a}	0.079±0.01^{3}	0^{b}	0^{b}	0^{b}	0^{b}
15	7.69	Phloridzin	C21H24O10	Glycosides	0.028±0.002^{c}	0.401±0.295^{b}	0.022±0.019^{c}	0^{c}	0.414±0.011^{b}	0^{c}	0.151±0.004^{c}	0.778±0.050^{a}	0.005±0.009^{c}
16	4.96	Loganin	C17H26O10	Terpenoids	0.033±0.006^{cd}	0.256±0.041^{a}	0.018±0.031^{cd}	0.009±0.016^{cd}	0.061±0.005^{bc}	0.106±0.009^{b}	0.266±0.026^{a}	0^{d}	0^{d}
17	5.36	Bilobalide	C15H18O8	Terpenoids	0.026±0.006^{b}	0.219±0.111^{b}	0.659±0.973^{b}	0.046±0.005^{b}	0^{b}	0.091±0.023^{b}	0^{b}	9.206±1.558^{a}	0.151±0.033^{b}
18	5.92	Complanatoside	C28H32O16	Glycosides	0.014±0.006^{bc}	0.014±0.024^{bc}	0.030±0.004^{b}	0.004±0.004^{c}	0.0273±0.002^{b}	0^{c}	0^{c}	0.156±0.007^{a}	0.001±0.002c
19	17.48	Curcumol	C15H24O2	Terpenoids	0.002±0.001c	2.656±0.414a	0.498±0.049c	0.113±0.013c	0.233±0.010c	1.168±0.034b	0.002±0.004c	0.006±0.011c	1.349±0.359b
20	10.44	Apigenin	C15H10O5	Flavonoids	0^{c}	0^{c}	0.089±0.023^{a}	0.082±0.007^{a}	0.040±0.012^{b}	0^{c}	0^{c}	0^{c}	0^{c}
21	6.11	Epicatechin gallate	C22H18O10	Carboxylic Acids	0^{b}	7.336±2.325^{a}	7.978±1.404^{a}	0.698±0.139^{b}	0.751±0.111^{b}	0.291±0.018^{b}	0^{b}	0^{b}	0.129±0.031^{b}
22	8.24	Spinosin	C28H32O15	Flavonoids	0^{b}	1.006±0.052^{b}	0.034±0.059^{b}	0^{b}	0^{b}	0.099±0.023^{b}	7.35±0.336^{b}	76.139±12.144^{a}	0.916±1.499^{b}

23	10.05	HeteroclitinD	C27H30O8	Hydrocarbons	0^{c}	0^{c}	2.912±0.699^{a}	0.904±0.262^{b}	0.915±0.042^{b}	3.140±0.471^{a}	0.779±0.025^{bc}	0^{c}	0.263±0.068^{bc}
24	15.6	Harpagide	C15H24O10	Glycosides	0.003±0.001^{b}	0.015±0.005^{b}	0.012±0.004^{b}	0^{b}	0^{b}	0.003±0.005^{b}	0.003±0.006^{b}	0.096±0.017^{a}	0^{b}
25	0.01	Ferulic Acid	C10H10O4	Carboxylic Acids	0^{b}	0^{b}	0.001±0.002^{b}	0^{b}	0.005±0.000^{a}	0^{b}	0^{b}	0^{b}	0^{b}
26	1.09	Malic acid	C4H6O5	Carboxylic Acids	0.213±0.035^{b}	0^{b}	0^{b}	0.176±0.018^{b}	0^{b}	0.371±0.020^{b}	0.907±0.059^{b}	5.533±1.069^{a}	0.191±0.033^{b}
27	1.12	5-Hydroxymethylfurfural	C6H6O3	Aldehydes	0^{c}	0.132±0.036^{a}	0^{c}	0.004±0.006^{c}	0.047±0.006^{b}	0^{c}	0^{c}	0^{c}	0^{c}
28	4.76	Bomyl acetate	C12H20O2	Carboxylic Acids	0^{a}	0^{a}	0.003±0.005^{a}	0.001±0.002^{a}	0.007±0.001^{a}	0.002±0.004^{a}	0.004±0.001^{a}	0.007±0.012^{a}	0^{a}
29	5.69	Gentiopicrin	C16H20O9	Glycosides	0^{b}	0^{b}	0^{b}	0^{b}	0^{b}	0.125±0.029^{b}	0.193±0.113^{b}	1.321±0.443^{a}	0.02±0.035^{b}
30	5.71	4-Hydroxycoumarin	C9H6O3	Benzopyrans	0^{b}	0^{b}	0^{b}	0.001±0.002b	0.004±0.000a	0^{b}	0^{b}	0^{b}	0^{b}
31	6.69	Benzoic acid	C10H12O5	Phenolic acids	0.001±0.002^{c}	0.043±0.011^{a}	0.013±0.003^{b}	0.001±0.001^{c}	0^{c}	0^{c}	0^{c}	0^{c}	0^{c}
32	7.34	Naringenin	C15H12O5	Flavonoids	0.023±0.001^{d}	0.203±0.030^{b}	0.114±0.009^{bcd}	0.056±0.012^{cd}	0.146±0.038^{bc}	0.013±0.023^{d}	0.077±0.006^{cd}	0.681±0.101^{a}	0.054±0.012^{cd}
33	7.83	Ellagic Acid	C14H6O8	Benzopyrans	0.095±0.031b	0.206±0.045a	0^{c}	0.163±0.221a	0^{c}	0.164±0.047a	0^{c}	0^{c}	0^{c}
34	8.29	Epimeredinoside A	C31H40O15	Others	0^{c}	0^{c}	0^{c}	0^{c}	0^{c}	0^{c}	0.040±0.03^{a}	0^{c}	0.014±0.002^{b}
35	8.49	Lobetyolin	C20H28O8	Hydrocarbons	0^{a}	0.043±0.074^{a}	0.017±0.030^{a}	0.005±0.003^{a}	0.009±0.002^{a}	0^{a}	0^{a}	0^{a}	0^{a}
36	8.58	Cichoric acid	C22H18O12	Carboxylic Acids	0^{a}	0^{a}	0^{a}	0^{a}	0.008±0.013^{a}	0^{a}	0.003±0.001^{a}	0^{a}	0^{a}

37	9.15	Dihydroquercetin	C15H12O7	Flavonoids	0[b]	0.427±0.171[a]	0.295±0.106[a]	0[b]	0[b]	0.039±0.068[b]	0[b]	0[b]	0.0147±0.003[b]
38	9.9	Benzoylpaeoniflorin	C30H32O12	Terpenoids	0[c]	0[c]	0[c]	0.756±0.017[b]	1.464±0.028[a]	0[c]	0[c]	0[c]	0[c]
39	10.59	Diosmetin	C16H12O6	Flavonoids	0[b]	0[b]	0[b]	0[b]	0[b]	0[b]	0[b]	0[b]	0.014±0.002[a]
40	11.92	Raspberry ketone	C10H12O2	Ketones	0.001±0.001[a]	0.001±0.001[a]	0.002±0.004[a]	0[a]	0[a]	0[a]	0.008±0.014[a]	0[a]	0[a]
41	16.01	Catharanthine	C21H24N2O2	Alkaloids	0.002±0.003[a]	0.008±0.014[a]	0[a]	0.004±0.001[a]	0[a]	0.004±0.006[a]	0[a]	0[a]	0[a]
42	16.23	Kaurenoic acid	C20H30O2	Terpenoids	0[c]	0[c]	0[c]	0.003±0.001[b]	0.001±0.002[bc]	0[c]	0.007±0.001[a]	0[c]	0.001±0.001[bc]

Supplementary Table S6. Antioxidant properties of the extracts from the newly grown leaves of WTPS ($n = 3 \pm SD$).

	ABTS (gTE/100g DW)	DPPH (gTE/100g DW)	FRAP (gTE/100g DW)	ORAC (gTE/100g DW)
P. jishanensis	38.158±0.66[b]	48.68±0.62[a]	20.89±0.72[b]	21.14±0.11[a]
P. qiui	29.00±0.77[c]	17.90±0.16[e]	17.79±1.44[c]	15.24±0.27[d]
P. decomposita	27.54±0.26[de]	17.59±0.15[e]	13.60±1.20[d]	13.25±0.32[ef]
P. ostii	27.96±0.74[d]	37.19±0.73[c]	26.51±0.21[a]	19.47±0.39[b]
P. rockii	29.69±0.27[c]	38.42±0.16[c]	17.67±1.04[c]	16.34±0.33[c]
P. lutea	49.62±0.13[a]	42.39±1.06[b]	26.23±0.10[a]	20.32±0.51[ab]
P. delavayi	27.05±0.17[de]	30.77±0.40[d]	16.05±0.30[c]	12.34±0.33[f]
P. ludlowii	27.00±0.13[de]	21.88±1.78[d]	13.14±0.40[d]	6.70±0.20[g]
P. potaninii	26.72±0.07[e]	36.66±0.57[c]	21.40±0.73[b]	13.38±0.33[e]

Different superscript letters in each column indicate significant differences ($P < 0.05$).

Supplementary Table S7. Minimum inhibitory concentrations of the newly grown leaves of nine wild tree peony species against the bacteria tested (*n* = 3).

Nine WTPS	Minimum inhibitory concentration (mg/mL)							
	S. aureus	*S.hemolytis-β*	*P. acnes*	*L. monocytogenes*	*P. aeruginosa*	*E. coli*	*S. enterica*	*P. vulgaris*
P. jishanensis	1.56	12.5	3.13	3.13	3.13	3.13	1.56	6.25
P. qiui	3.13	6.25	6.25	3.13	3.13	6.25	1.56	6.25
P. decomposita	1.56	6.25	3.13	3.13	6.25	6.25	6.25	6.25
P.ostii	0.78	12.5	3.13	1.56	6.25	3.13	6.25	6.25
P.rockii	0.78	12.5	3.13	0.78	12.5	6.25	1.56	3.13
P.lutea	1.56	12.5	6.25	0.78	12.5	12.5	3.13	3.13
P. delavayi	1.56	25	6.25	6.25	12.5	12.5	3.13	6.25
P. ludlowii	3.13	12.5	12.5	6.25	6.25	12.5	3.13	12.5
P. potaninii	1.56	12.5	3.13	3.13	6.25	12.5	6.25	6.25
Kanamycin	0.16	0.08	2.5	0.31	2.5	1.25	1.25	0.16

Supplementary Table S8. Absorbance values of different concentrations of gallic acid standard solutions at 765 nm.

Absorbance values of different concentrations of gallic acid standard solutions at 765 nm										
Concentrations of gallic acid standard solutions (mg/ml)	0	0.01	0.02	0.03	0.04	0.05	0.06	0.07	0.08	0.09
Absorbance values	0	0.167	0.2457	0.3463	0.4034	0.572	0.6661	0.77	0.8764	0.9663

Supplementary Table S9. Absorbance values of different concentrations of rutin standard solutions at 506 nm.

Absorbance values of different concentrations of rutin standard solutions at 506 nm										
Concentrations of rutin standard solutions (mg/ml)	0	0.01	0.02	0.03	0.04	0.05	0.06	0.07	0.08	0.09
Absorbance values	0	0.1109	0.1645	0.205	0.2513	0.3004	0.3594	0.4035	0.4297	0.48

Supplementary Figures

Supplementary Figure S1. DIZ of four Gram-positive bacteria after the application of peony leaf extracts at a concentration of 0.2 mL/cup. Bars = 10 mm. (A) *S. aureus*. (B) *S. hemolytis-β*. (C) *P. acnes*. (D) *L. monocytogenes*. (I) *P. jishanensis*. (II) *P. qiui*. (III) *P. decomposita*. (IV) *P. ostii*. (V) *P. rockii*. (VI) *P. lutea*. (VII) *P. delavayi*. (VIII) *P. ludlowii*. (IX) *P. potaninii*. (1) Replicate 1. (2) Replicate 2. (3) Replicate 3. (4) Negative controls (Methanol). (5) Positive controls (Kanamycin).

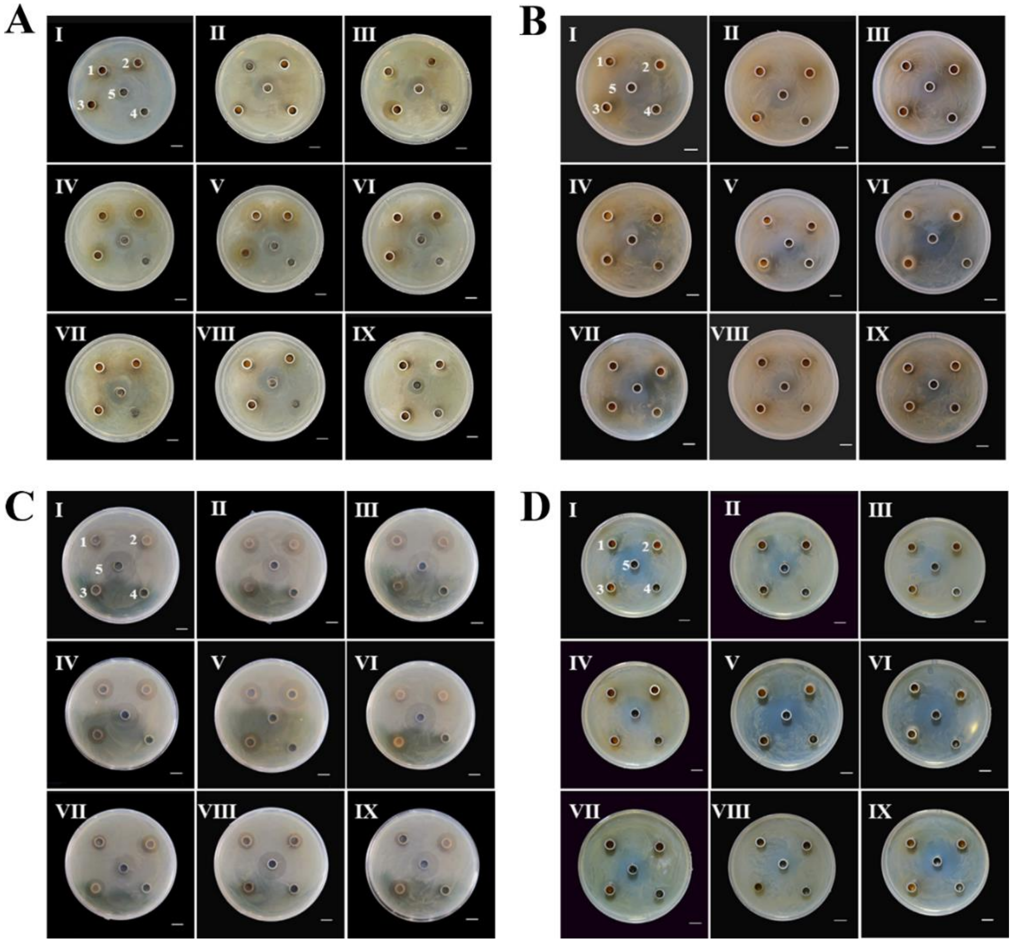

Supplementary Figure S2. DIZ of four Gram-negative bacteria after the application of peony leaf extracts at a concentration of 0.2 mL/cup. Bars = 10 mm. (A) *P. aeruginosa*. (B) *E. coli*. (C) *P. vulgaris*. (D) *S. enterica*. (I) *P. jishanensis*. (II) *P. qiui*. (III) *P. decomposita*. (IV) *P. ostii*. (V) *P. rockii*. (VI) *P. lutea*. (VII) *P. delavayi*. (VIII) *P. ludlowii*. (IX) *P. potaninii*. (1) Replicate 1. (2) Replicate 2. (3) Replicate 3. (4) Negative controls (Methanol). (5) Positive controls (Kanamycin).

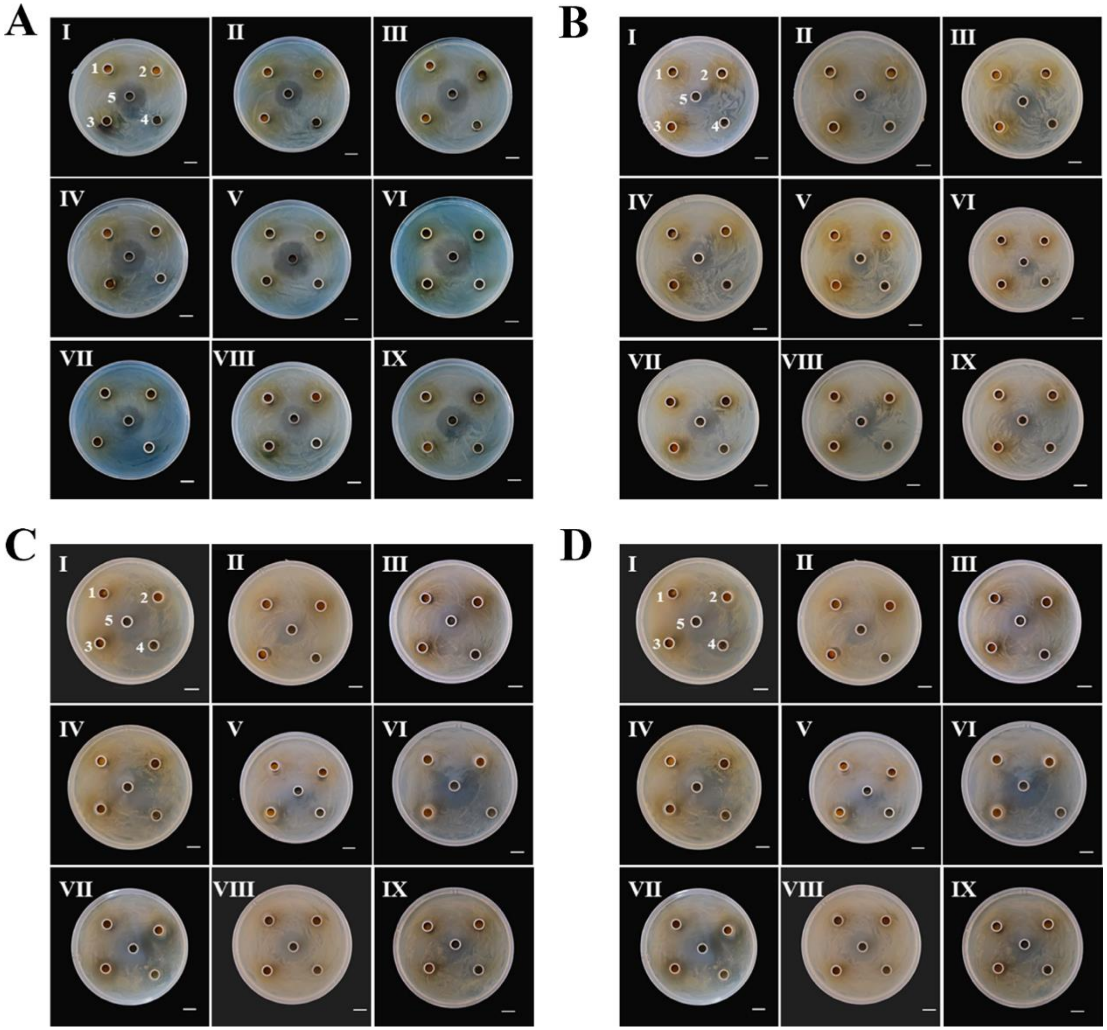

Supplementary Figure S3. The regression curves with different concentrations of gallic acid standard solutions

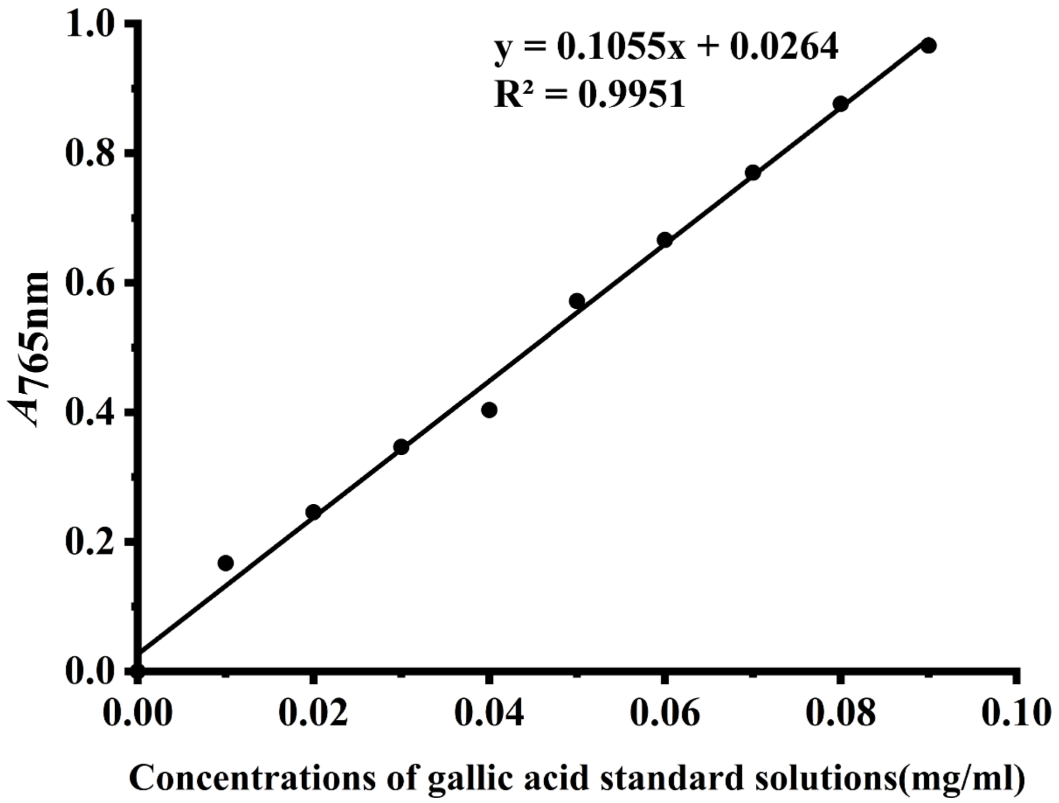

Supplementary Figure S4. The regression curves with different concentrations of rutin standard solutions

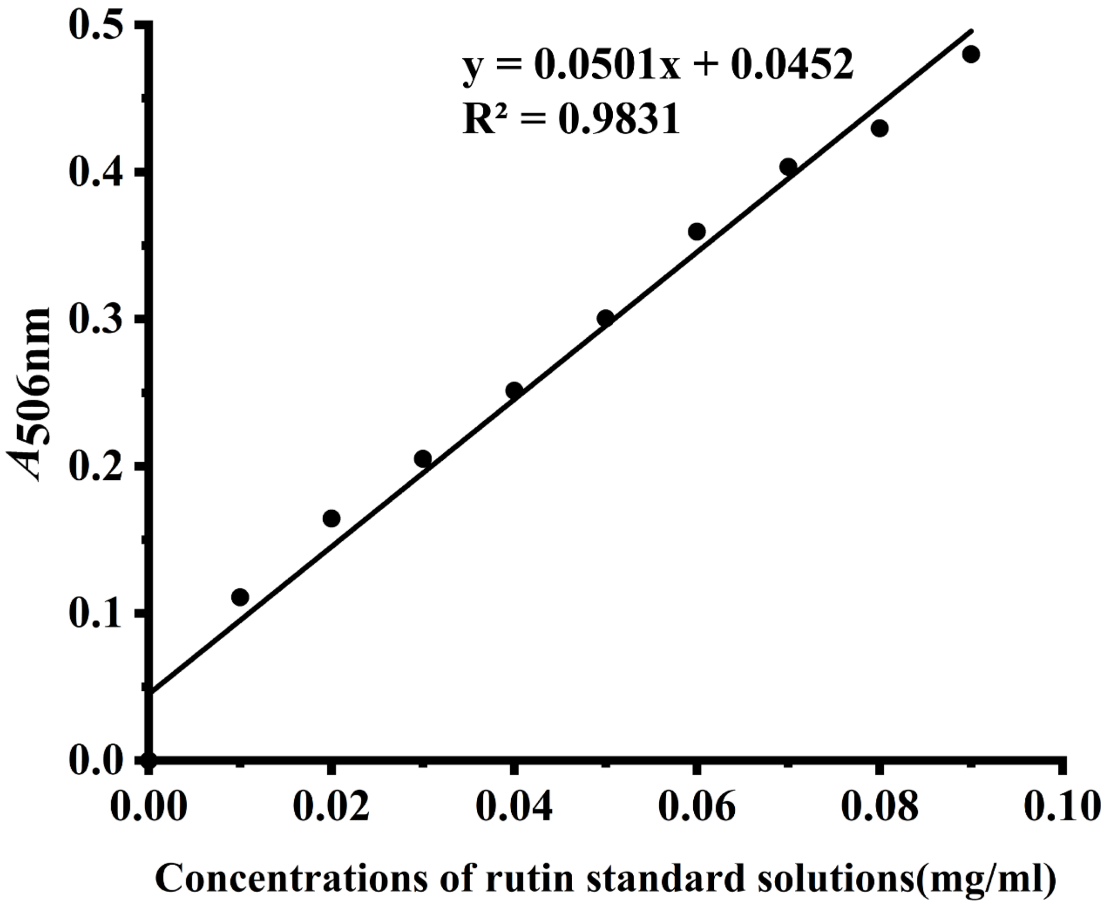

Supplement: Supplementary file 1 [file ijms-24-10609-s001.zip › ijms-2452206-supplementary.pdf]
